# Spatial patterns of phylogenetic diversity

**DOI:** 10.1111/j.1461-0248.2010.01563.x

**Published:** 2011-02

**Authors:** Hélène Morlon, Dylan W Schwilk, Jessica A Bryant, Pablo A Marquet, Anthony G Rebelo, Catherine Tauss, Brendan J M Bohannan, Jessica L Green

**Affiliations:** 1 Center for Ecology and Evolutionary Biology, University of OregonEugene, OR, USA; 2 Texas Tech UniversityLubbock, TX, USA; 3 Massachussetts Institute of TechnologyCambridge, MA, USA; 4 Center for Advanced Studies in Ecology and Biodiversity and Departamento de Ecología, Pontificia Universidad Católica de ChileSantiago, Chile; 5 Instituto de Ecología y BiodiversidadCasilla 653, Santiago, Chile; 6 Santa Fe InstituteSanta Fe, NM, USA; 7 South African National Biodiversity InstituteKirstenbosch, South Africa; 8 School of Plant Biology, University of Western AustraliaPerth, Australia

**Keywords:** Community phylogenetics, conservation, distance–decay relationship, evolutionary history, Mediterranean-type ecosystems, phylogenetic beta-diversity, phylogenetic diversity, spatial scaling, species–area relationship

## Abstract

Ecologists and conservation biologists have historically used species–area and distance–decay relationships as tools to predict the spatial distribution of biodiversity and the impact of habitat loss on biodiversity. These tools treat each species as evolutionarily equivalent, yet the importance of species' evolutionary history in their ecology and conservation is becoming increasingly evident. Here, we provide theoretical predictions for phylogenetic analogues of the species–area and distance–decay relationships. We use a random model of community assembly and a spatially explicit flora dataset collected in four Mediterranean-type regions to provide theoretical predictions for the increase in phylogenetic diversity – the total phylogenetic branch-length separating a set of species – with increasing area and the decay in phylogenetic similarity with geographic separation. These developments may ultimately provide insights into the evolution and assembly of biological communities, and guide the selection of protected areas.

## Introduction

Community ecologists and conservation biologists are increasingly analysing phylogenetic information and community data in tandem (Webb *et al.* 2002; Purvis *et al.* 2005; Forest *et al.* 2007; Cavender-Bares *et al.* 2009; Vamosi *et al.* 2009; Winter *et al.* 2009; Devictor *et al.* 2010). For example, the phylogenetic structure of local communities is compared with that of larger species pools to understand the processes driving community assembly (Webb *et al.* 2002; Heard & Cox 2007; Graham & Fine 2008; Cavender-Bares *et al.* 2009; Kraft & Ackerly 2010). Similarly, phylogenetic diversity (PD) is mapped across landscapes to select conservation areas that optimize the preservation of evolutionary history (Rodrigues & Gaston 2002; Ferrier *et al.* 2007; Forest *et al.* 2007; Winter *et al.* 2009; Devictor *et al.* 2010).

Despite the growing interest in PD, spatial biodiversity research has historically been centred on patterns of species diversity. Hundreds of publications have documented the species–area relationship (Preston 1962; Rosenzweig 1995), which describes the increase in species richness with geographic area. Touted as one of the few general laws in ecology, the species–area relationship has been crucial to the development of ecological theory (Preston 1962; MacArthur & Wilson 2001; Chave *et al.* 2002) and for estimating extinction risk in the face of environmental change (Pimm & Askins 1995; Guilhaumon *et al.* 2008). Similarly, analytical characterizations of the curve describing how the similarity in species composition between two communities decays with the geographic distance separating them (the distance–decay relationship) have been used to infer the relative importance of dispersal limitation and environmental filtering in explaining patterns of diversity (Preston 1962; Nekola & White 1999; Chave & Leigh 2002; Condit *et al.* 2002; Morlon *et al.* 2008), and to predict the complementarity of sites within reserve networks (Ferrier *et al.* 2007).

In contrast to the decades of research on the spatial scaling of species diversity, research on the spatial scaling of PD remains in its infancy. Empirical observations of the increase of PD with area (Rodrigues & Gaston 2002), and of the decay in phylogenetic similarity with geographic or environmental distance (Chave *et al.* 2007; Hardy & Senterre 2007; Bryant *et al.* 2008) have recently emerged. However, there have been no attempts to generalize the shape or mathematical form of these diversity patterns. This is a major gap, given that patterns explicitly incorporating information on evolutionary history will likely be more powerful than patterns that do not (such as the species–area and distance–decay relationships) for testing, and estimating parameters of, biodiversity theory (Jabot & Chave 2009). Furthermore, phylogeny-based spatial patterns are needed for setting conservation priorities aimed at protecting evolutionary history in a spatial context (Rodrigues & Gaston 2002; Purvis *et al.* 2005; Ferrier *et al.* 2007; Winter *et al.* 2009; Devictor *et al.* 2010).

There are three main determinants to the spatial scaling of PD: the spatial scaling of species diversity, the phylogenetic tree describing the evolutionary history of these species and their position in the phylogeny. In turn, these three components are driven by multiple evolutionary and ecological processes, including speciation and extinction, dispersal limitation, environmental filtering, and intra- and inter-specific interactions. Recently, much focus has been given to the third component (the position of co-occurring species in a phylogeny), often referred to as community phylogenetic structure. Phylogenetic structure measures the extent to which species assemblages deviate from random assemblages and has been used as a tool to infer the processes underlying community assembly (Webb *et al.* 2002; Heard & Cox 2007; Graham & Fine 2008; Cavender-Bares *et al.* 2009; Kraft & Ackerly 2010).

**Figure 1:**
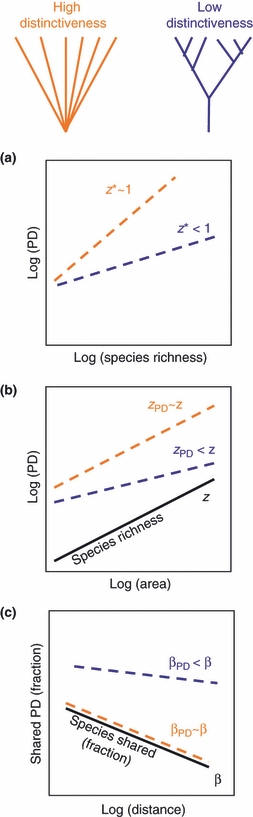
Conceptual figure illustrating, under the random community assembly model, the expected effect of phylogenetic tree shape on the relationship between (a) phylogenetic diversity (PD) and species richness, (b) PD and habitat area and (c) phylogenetic similarity and geographic distance. Here the PD of a set of species is measured as the phylogenetic branch-length joining all species in the set to the root. Star-like phylogenetic trees (with high distinctiveness, in orange) are characterized by steep species–PD curves (slope z* ∼ 1). Phylogenies with decreasing distinctiveness (in blue) have shallower species–PD curves, resulting in shallower PD–area curves, and shallower phylogenetic distance–decay curves.

In this article, we use a model where species are randomly assembled with respect to phylogeny to derive predictions for the spatial scaling of PD in the absence of phylogenetic structure. This reduces the task to two well-studied problems, usually considered separately in the literature: modelling spatial patterns of species diversity, and modelling cladogenesis. Under the random assembly model, the link between species-based diversity patterns and the spatial scaling of PD is given by the species–PD curve, which describes how PD increases with an increasing number of species randomly sampled from a given phylogeny (Fig. 1). The species–PD curve has been studied in conservation, as it provides estimates for the potential loss of PD due to extinctions (Nee & May 1997; Heard & Mooers 2000; Diniz-Filho 2004; Purvis *et al.* 2005; Soutullo *et al.* 2005). The species–PD curve is a function only of the underlying phylogeny, not the spatial configuration of communities, and can thus be studied using models of cladogenesis developed in macroevolution (Nee & May 1997; Heard & Mooers 2000; Nee 2006; Morlon *et al.* 2010).

We first derive testable predictions of how PD increases with geographic area, and how phylogenetic similarity decays with geographic distance. We then demonstrate the validity of these predictions in nature using a spatially explicit dataset collected in the four Mediterranean-type ecosystems of Australia, California, Chile and South Africa. Finally, we discuss implications of our study for community ecology, biogeography and conservation.

## Materials and Methods

### Mediterranean flora data

Data for woody angiosperms in the Mediterranean climate shrublands of Australia, California, Chile and South-Africa were collected between April and December 2006 (see Appendix S1 of Supporting Information). On each continent, we sampled 30 quadrats (120 quadrats total), separated by geographic distances ranging from 20 m (adjacent) to 170 km (Appendix S1). Within each quadrat, presence/absence data were recorded at the 2.5 × 2.5, 7.5 × 7.5 and 20 × 20 m scales, except in California where data were only recorded at the 20 × 20 m scale (Fig. S1). We sampled in a relatively homogeneous flora and environment within each Mediterranean-type ecosystem. Specifically, plots were sampled on the same parent material, and slope, aspect and fire history were kept as constant as possible. A total of 538 species encompassing 254 genera and 71 families were identified: 177 in the Australian kwongan, 27 in the Californian chaparral, 44 in the Chilean matorral and 290 in the South African fynbos (Fig. S2).

### Phylogenetic construction

We used a megatree approach to construct a hypothesized dated phylogenetic tree for the species present in our dataset (Webb & Donoghue 2005). We first built an angiosperm backbone tree by supplementing the Phylomatic2 phylogenetic data repository (http://svn.phylodiversity.net/tot/trees/), which is based on resolutions from the Angiosperm Phylogeny Group, with additional data found in the literature (Appendix S8). We then grafted the 538 species in our dataset onto the backbone tree; the resulting phylogeny is thereafter referred to as the ‘full phylogeny’. To assign branch-lengths, we spaced undated nodes evenly between dated ones using a slightly modified version of the Branch Length Adjuster (BLADJ) algorithm, as described next (Webb *et al.* 2008; Cam Webb, personal communication; code available at: http://www.schwilk.org/research/data.html). The full phylogeny included terminal nodes that were not species. Specifically, the full phylogeny had 874 terminal nodes, 538 of which corresponded to the species in the dataset; the 336 remaining terminal nodes were families or genera in the backbone tree with no representative in the dataset. To ensure that we included during the branch-length assignment procedure all clades for which a node age estimate was available (Wikström *et al.* 2001), we fixed the terminal nodes corresponding to family or genera to their estimated ages before running the BLADJ algorithm. The phylogeny of the entire dataset (the ‘combined phylogeny’) was then obtained by removing nodes with no representative in the dataset. Individual phylogenies for each of the four regional datasets (the ‘regional phylogenies’) were obtained by pruning to the corresponding set of species (Appendix S1).

### Phylodiversity metrics

There are several ways to measure PD within and among communities (see Lozupone & Knight 2008; Vamosi *et al.* 2009; Cadotte *et al.* 2010 for reviews). Given that our goal was to build spatial phylogenetic patterns readily comparable with the species-based species–area and distance–decay relationships, we chose metrics that most closely capture the notion of total amount of evolutionary history contained within, and shared between, communities. In addition, we excluded abundance-based metrics (Chave *et al.* 2007; Cadotte *et al.* 2010) because we collected incidence data only.

We quantified the PD of a given sample (alpha diversity) as the total phylogenetic branch-length joining the basal node (here the angiosperm node) to the tips of all the species in the sample (‘PD’; Faith 1992). This metric is proportional to species richness for a star phylogeny (i.e. a phylogeny where species share no branch-length), rendering comparisons with the traditional species–area relationship possible. PD has the added advantage of being the phylodiversity metric of choice in conservation research (Faith 1992; Nee & May 1997; Rodrigues & Gaston 2002; Purvis *et al.* 2005; Forest *et al.* 2007; Winter *et al.* 2009). Diversity metrics based on pairwise taxon distances between species (Chave *et al.* 2007; Hardy & Senterre 2007) are not proportional to species richness for a star phylogeny, and they are rarely used for conservation purposes (Cadotte *et al.* 2010). Faith’s PD retains the root of the species pool phylogeny, and this may reduce the variance in PD among samples (Crozier 1997; Crozier *et al.* 2005). However, as illustrated next, including the root is useful for constructing metrics of phylogenetic beta-diversity.

We quantified the phylogenetic similarity between two communities (an inverse measure of phylogenetic beta-diversity) with the incidence-based PhyloSor index χ_PD_, which measures the PD shared between communities (noted PD_1,2_) divided by the average PD in each community: 
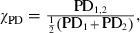
 where PD_1_ and PD_2_ represent the PD of each community (Bryant *et al.* 2008). Equivalently, 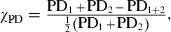 where PD_1+2_ is the PD of the two communities combined. This index is closely related to indices suggested by Ferrier *et al.* (2007) to measure complementarity for conservation purposes, as well as to the Unifrac metric, widely used in microbial ecology research (Lozupone & Knight 2008). For a star phylogeny, the Phylosor index reduces to the Sorenson index of similarity, which is commonly used to characterize distance–decay relationships (Preston 1962; Nekola & White 1999; Morlon *et al.* 2008). If the root is not retained in the calculation of PD, PD_1_ + PD_2_ − PD_1+2_ can take negative values (e.g. if communities 1 and 2 are composed of distinct, distantly related clades), which is biologically unrealistic.

### Random assembly hypothesis

Our approach to deriving predictions for the increase of PD with area and the decay in phylogenetic similarity with geographic distance is to assume that the curves describing the increase in species richness with area and the decay in species similarity with geographic distance are known. This approach allows leveraging decades of research on the species–area and distance–decay relationships to understand how PD is distributed spatially.

Once species richness and species spatial turnover are known across a landscape, there are several ways to map a given phylogeny onto this landscape. We chose the simplest approach, which is to randomly assign a tip to each species in the landscape. This random assembly model is increasingly being used in community phylogenetics and consists of randomizing the position of species on a phylogeny while keeping species richness and turnover constant (Bryant *et al.* 2008; Graham *et al.* 2009). This model corresponds to the hypothesis that species are randomly assembled with respect to phylogeny within and across communities. Here, our primary interest in using this model is to provide a tractable theoretical approach for investigating spatial PD patterns.

To evaluate the validity of the random assembly hypothesis in our data, we tested for deviations from the random assembly model at each spatial scale within each 20 × 20 m plot. To do this, we compared the total PD of the observed communities with that of communities composed of the same number of species assembled by random sampling from each regional phylogeny. We also compared the observed phylogenetic similarity between pairs of communities, sampled at the 20 × 20 m scale, with that of communities composed of, and sharing, the same number of species assembled by random sampling from each regional phylogeny. In other words, we randomized species across the tips of regional phylogenies while holding alpha- and beta-diversity constant (Bryant *et al.* 2008; Graham *et al.* 2009; Appendix S2).

### Spatial PD theory predictions

Our spatial phylogenetic theory predictions build on the random assembly hypothesis and the observation that, if there exists a consistent relationship between PD and an increasing number of species randomly sampled in a phylogeny (the species–PD curve), then spatial patterns of PD may be deduced from this curve (Fig. 1). We obtained species–PD curves for each of the four regional phylogenies and for the combined phylogeny by randomly sampling an increasing number of species in each phylogeny, 100 times at each richness value. For comparison with previous studies, we fitted a logarithmic function to the observed species–PD curves, which is the only published analytical prediction for species–PD curves we are aware of (equation 1 in Nee & May 1997). Sensitivity analyses were conducted to evaluate the influence of polytomies and the BLADJ branch-length assignment procedure on the observed species–PD curve (Appendix S3).

Using the best-fit functional form for the species–PD curve in our data, we derived theoretical predictions for the increase of PD with area and the decay of phylogenetic similarity with geographic distance under the random assembly hypothesis. To test the accuracy of these predictions, we compared the predicted PD–area relationship and decay in phylogenetic similarity with geographic distance in each region with the 95% confidence envelopes of the curves obtained by simulations of the random assembly process (Appendix S4).

We also tested the ability of the random assembly process to reproduce the observed spatial PD patterns in each region. To do this, we computed the observed PD–area relationship by quantifying PD at the 2.5 × 2.5, 7.5 × 7.5 and 20 × 20 m scales in each of the 30 quadrats (except in California where data were only collected at the 20 × 20 m scale), and the decay in phylogenetic similarity with geographic distance by quantifying χ_PD_ between each pair of communities (435 pairs in each regional dataset) at the 20 × 20 m scale. We compared the observed relationships with the 95% confidence envelopes of the curves obtained by simulations of the random assembly process (Appendix S4).

All analyses were carried out using the Picante software package implemented in R (Kembel *et al.* 2010).

## Results

### Random assembly hypothesis

Within each of the four Mediterranean flora datasets, most communities did not significantly deviate from the random assembly model (Fig. S3). Similarly, the fraction of PD shared between most pairs of communities within each dataset was not significantly different than that expected by chance given their species richness and fraction of species shared (Fig. S4). The dataset was thus ideal for testing predictions about the increase in PD with area, and the decay in phylogenetic similarity with geographic distance, under the random assembly model.

### Species–PD curves and the shape of regional phylogenies

**Figure 2:**
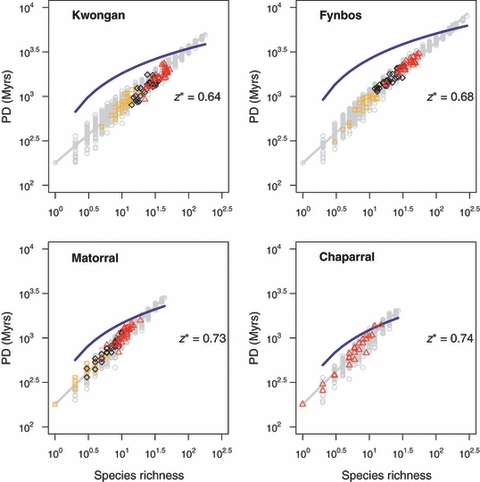
Species–phylogenetic diversity (PD) curves in Mediterranean-type ecosystems. The grey circles report, for each value of species richness (S), the PD of 100 communities obtained by randomly sampling S species across the tips of each phylogeny (species–PD relationship). This relationship is well fit by a power law in the four phylogenies (eqn 1, plain grey line). In particular, the power-law fit is much better than the best-fit logarithm (in blue). The intercept of both fits is constrained by the age of the most recent common ancestor, T_0_. The species–PD curve corresponding to the combined dataset is also power law, with z* = 0.71 (Figure S3). Coloured data points correspond to actual communities. Orange squares: communities sampled at the 2.5 × 2.5 m scale; black diamonds: communities sampled at the 7.5 × 7.5 m scale; red triangles: communities sampled at the 20 × 20 m scale. Most communities are not significantly different from randomly assembled communities (see Appendix S2 for details).

When an increasing number of species (S) were randomly drawn in each regional phylogeny, the corresponding increase in PD (species–PD curve) was well approximated by a power-law relationship (Fig. 2). This pattern also held for the combined phylogeny (Fig. S5). The power-law shape was robust to the presence of polytomies and the branch-length assignment procedure (Appendix S3), suggesting that it was not an artefact of the method of phylogenetic construction. In particular, the power law provided a much better fit to the species–PD curve than the logarithmic function (Fig. 1 and Appendix S3).

A power-law species–PD relationship takes the form: 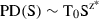
(1) with the normalization constant given by the age T_0_ of the most recent common ancestor in the phylogeny. This expression provides an expectation for the PD of a community containing S species, under the random assembly hypothesis. This expression also characterizes the species–PD curve by a single exponent z* (z* ≤ 1) which captures information about the phylogenetic distinctiveness of species (i.e. how evolutionarily unique species are relative to one another within a phylogeny; Vane-Wright *et al.* 1991; Fig. 1a). High z* values correspond to trees with high distinctiveness (typically, trees with long terminal branches and high imbalance), while low z* values correspond to trees with low distinctiveness (i.e. trees with short terminal branches and low imbalance). We found z* values ranging from above 0.7 in the matorral and chaparral, to 0.68 in the fynbos and 0.64 in the kwongan. z* values were slightly lower in the kwongan and fynbos due to the presence of closely related species in floras that radiated recently (Richardson *et al.* 2001).

We used the power-law species–PD curve to characterize the relationship between phylogenetic distinctiveness, the spatial distribution of species and spatial patterns of PD (Fig. 1). We used the power law because it is a convenient mathematical approximation, and also because it may be general to many phylogenetic trees. We observed a power-law relationship in all four datasets we studied. This consistency across datasets suggests generality, given that less than 25% of PD was shared between any two datasets. In cases where the power-law approximation is not accurate, our approach may be readily modified to account for alternative characterizations of species–PD curves (see next).

### Increase of PD with area

**Figure 3:**
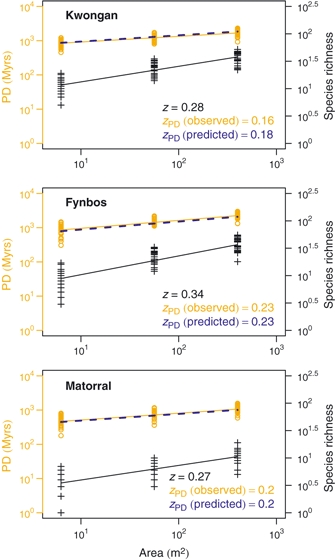
The increase of phylogenetic diversity (PD) with area in Mediterranean-type ecosystems. The observed PD–area relationship (in orange: circles, data; line, power-law fit) is well approximated by an expectation (eqn 4, in blue) obtained by simple power transformation of the classical species–area relationship (in black: crosses, data; line, power-law fit). The power-law exponent z_PD_ of the PD–area relationship is well approximated by the product of the power-law exponent of the species–area relationship z and the power-law exponent of the species–PD relationship z*. PD increases with habitat area at a slower pace than species, and the difference is the largest in floras where species are the least phylogenetically distinct (i.e. in the kwongan and fynbos).

Under the hypothesis that species assemblages are random with respect to phylogeny at each spatial scale, and assuming the power-law scaling between PD and species richness (eqn 1), the expected PD contained in a sample of area A is given by: 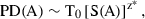
(2) where S(A) is the expected number of species contained in a sample of area A (the species–area relationship). A classic form of the species–area relationship is the power law: 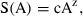
(3) where c is a normalization constant, and z typically varies around the value of 0.25 (Rosenzweig 1995). While variations around the power-law species–area curve are common (Guilhaumon *et al.* 2008), the power law yielded a good description of the increase of species richness with area in our data (Fig. 3). The shape of the PD–area relationship may then be characterized by a power law with exponent z_PD_, the product of the power-law exponent z of the species–area relationship and of the power-law exponent z* of the species–PD curve: 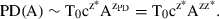
(4)

This equation provides an expectation for the PD of a community spanning an area A, under the random assembly hypothesis. The power-law PD–area curve is shallower than the species–area curve by a factor z*, showing that PD increases with area at a slower pace than species richness (Fig. 1b). The power-law species–area and PD–area curves imply that if a fraction x of a given area is preserved, a fraction x^z^ of species is preserved (eqn 3), corresponding to a fraction x^zz*^of preserved PD (eqn 4). Equation 4 may be used to provide estimates for the loss of PD with habitat loss (see Appendix S5 for estimates in Mediterranean-type ecosystems).

The PD–area relationships observed in the three Mediterranean-type ecosystems were well described by eqn 4, which is based on power-law scaling relationships (Figs 3 and S10). Other forms of the species–PD curve and species–area relationship may better describe other systems. This would yield different shapes for the PD–area relationship that could be derived using a similar approach (Appendix S6).

### Decay of phylogenetic similarity with geographic distance

To derive expectations for the decay in phylogenetic similarity with geographic distance, we maintained our assumption that communities are randomly assembled with respect to phylogeny. Using the power-law scaling between PD and species richness, we found (Appendix S7) that the expected fraction of PD shared between two communities, each spanning an area A, and separated by geographic distance d is given by: 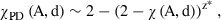
(5) where 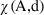 is the expected Sorensen index of similarity. This equation confirms, as expected intuitively, that communities share a greater fraction of PD than species 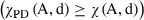.

**Figure 4:**
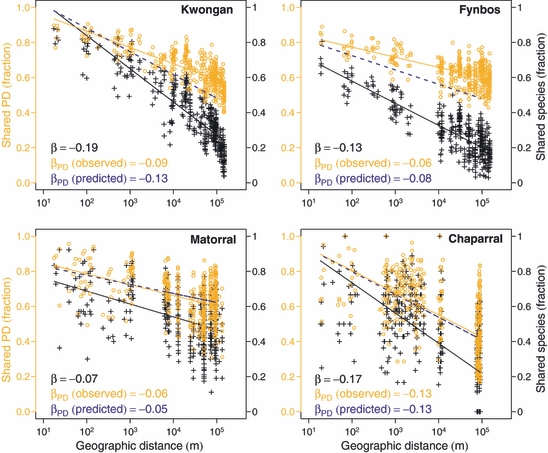
Decay in phylogenetic similarity with geographic distance within Mediterranean-type ecosystems. The observed phylogenetic distance–decay relationship (in orange: circles, data; line: logarithmic fit) can be approximated by expectations (eqn 6, in blue) obtained by simple transformation of the classical distance–decay relationship for species turnover (in black: crosses, data; line: logarithmic fit). The rate of decay in phylogenetic similarity (β_PD_) is significant in all four datasets (mantel test, *P*< 0.001). This rate is lower than the rate of decay in taxonomic similarity (β), and the difference is the largest in floras where species are the least phylogenetically distinct (i.e. in the kwongan and fynbos).

To further formalize the scaling between phylogenetic similarity and geographic distance, we assumed a logarithmic model for the species-based distance–decay relationship of the form 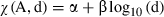. We chose the logarithmic model because it provided a good fit to our data (Fig. 4). The logarithmic model has been observed in tropical forest communities, and has the additional value of being the predicted beta-diversity pattern under the neutral theory of biodiversity (Chave & Leigh 2002; Condit *et al.* 2002). With this model, and under the random assembly hypothesis, the expected shape of the phylogeny-based distance–decay relationship may also be described by a logarithmic function (Appendix S7): 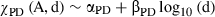
(6) with 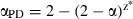 and 
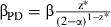
.

Equation 6 provides an expectation for the fraction of PD shared between two communities spanning an area A and separated by a distance d. Although deviations from this equation occurred (e.g. in the kwongan and fynbos; Figs 4 and S11), the equation yielded a good description of the data in the matorral and chaparral. Equation 6 suggests that the rate of decay in phylogenetic similarity (β_PD_) is less than the rate of decay in species similarity (β). This suggests that, within reserve networks, a greater spatial separation between protected sites will be required to preserve PD relative to the spatial extent required to preserve species richness.

Across Mediterranean-type ecosystems, no species were shared. The ecosystems that have been historically connected by landmasses and/or share geological attributes (e.g. California–Chile, Australia–South Africa and Chile–South Africa) were more phylogenetically similar (respective χ_PD_ values obtained by pulling all species within each dataset: 0.28, 0.26, 0.20) than Mediterranean-type systems that have been separated by oceans for longer time periods and/or are geologically very distinct (e.g. Australia–Chile, Australia–California and California–South Africa, χ_PD_ value ∼ 0.18 for all three pairs). When no species are shared and under the random model of community assembly, eqn 4 suggests that the phylogenetic similarity between the two communities equals 2 − 2^z*^. The phylogenetic similarity between datasets was much lower than this expectation, reflecting dispersal limitation across continents acting over evolutionary time scales.

## Discussion

Although there has been an explosion of community phylogenetics papers in the last few years, no study has clearly identified the mathematical form of spatial PD patterns. In this article, we provide theoretical predictions for the increase of PD with area and the decay in phylogenetic similarity with geographic distance under a model of random assembly from the regional species pool. These predictions have implications for conservation and for our understanding of how communities assemble.

In the future, conservation planners will likely leverage spatial models of PD to inform policy. The PD–area relationship, for example, can be used to estimate the potential loss of PD following habitat loss. Phylogenetically informed conservation research has primarily been focused on global-scale PD loss (Nee & May 1997), but the loss of PD at smaller spatial scales is of equal concern (e.g. Rodrigues & Gaston 2002; Forest *et al.* 2007; Winter *et al.* 2009; Devictor *et al.* 2010). For example, conservation strategies are often implemented at the level of geopolitical units interested in preserving regional evolutionary heritage and associated biological attributes of ethical, medical or economic value (Mooers & Atkins 2003; Purvis *et al.* 2005; Soutullo *et al.* 2005). Losing PD at any scale can lead to a reduced potential for communities to respond to changing environmental conditions, through a reduction of genetic diversity (Purvis *et al.* 2005).

Our derivation of the PD–area relationship shows that diversity depends on habitat area less strongly when measured as total phylogenetic branch-length vs. species richness. Although this may seem intuitive, a study by Rauch & Bar-Yam (2005), carried out in the context of population genetics, suggested the opposite pattern. This discrepancy is explained by the implicit assumption in Rauch and Bar-Yam’s study that a genealogy remaining in a preserved area following habitat loss evolved solely in the preserved area. In contrast, our derivations acknowledge that a phylogeny observed after habitat loss is a sample of a phylogeny evolved in a larger area. Our derivations will thus provide more realistic estimates of PD loss with habitat loss.

Patterns of phylogenetic beta-diversity also have implications for conservation (Ferrier *et al.* 2007; Winter *et al.* 2009; Devictor *et al.* 2010). Communities share a greater fraction of PD than species (eqn 5). This suggests, as expected intuitively, that a single isolated area is more efficient in preserving PD than species richness. On the other hand, the phylogenetic similarity between communities decays with geographic distance at a slower pace than the similarity in species composition (eqns 5 and 6), such that larger distances between protected sites are needed to preserve PD relative to species diversity. In practice, as habitat degradation proceeds, conservation planners might have to choose between protecting distant but degraded sites vs. proximate but pristine ones. If degraded sites have lost their phylogenetic uniqueness, as can result from invasions (Winter *et al.* 2009), the beneficial effect of separating sites spatially needs to be compared with the beneficial effect of preserving the most unique species in pristine areas.

To make predictions about spatial PD patterns, we used species–PD curves. In our data, we found that species–PD curves were accurately modelled by power laws. This was not expected *a priori*: previous research predicted a logarithmic species–PD curve (equation 1 in Nee & May 1997). The logarithmic curve was not supported by our data, and there are multiple reasons to expect that it will not characterize empirical phylogenies. The logarithmic species–PD curve arises from Hey’s model of cladogenesis, which is known to produce phylogenies with much shorter terminal branches than empirical phylogenies (Hey 1992). As terminal branches get longer than expected under Hey’s model, species–PD curves become steeper than the logarithm and they tend toward a power-law function. Many phylogenies in nature have long terminal branches, as suggested by the preponderance of empirical phylogenies with negative values of the gamma statistic (negative gamma values reflect long terminal branches; Pybus & Harvey 2000). In addition, sampled phylogenies (e.g. continental or regional phylogenies) have fewer nodes towards the present than global-scale phylogenies, resulting in longer terminal branches (Pybus & Harvey 2000). Hence, the power-law approximation may be general to species–PD curves for a variety of taxonomic groups, sampled at a variety of spatial scales.

Our empirical evidence for power-law species–PD curves, rather than a logarithmic function, is relevant to seminal work linking species extinction and the loss of evolutionary history (Nee & May 1997; Heard & Mooers 2000). Nee & May (1997) suggested that PD is highly robust to random extinctions, based on the logarithmic shape of species–PD curves. This study has been criticized on the basis that extinctions are not random with respect to phylogeny (Heard & Mooers 2000; Purvis *et al.* 2000). However an even greater source of bias may come from the assumed shape for species–PD curves. The power-law shape observed in this study suggests that PD is not robust to extinctions, even under random loss. Intuitively, this increased loss of PD with extinction stems from the fact that species are much more evolutionarily distinct than expected under Hey’s model.

In addition to assuming a power law species–PD curve, we assumed a random community assembly model. Within Mediterranean-type ecosystems, our data did not depart from this model. This absence of phylogenetic structure was likely a consequence of sampling in relatively homogeneous floras and environments, and at relatively small spatial scales. Deviations from the random assembly model are common in nature (Cavender-Bares *et al.* 2009; Vamosi *et al.* 2009) and have been reported in Mediterranean-type ecosystems (Proches *et al.* 2006; Forest *et al.* 2007).

A wide array of processes can lead to deviations from phylogenetic patterns predicted under the random assembly model. In turn, these deviations might offer insight into ecological and evolutionary processes. Within scales where species are not limited by their capacity to disperse, and under the hypothesis of trait conservatism, communities often switch from phylogenetic overdispersion at the smallest spatial scales (i.e. co-occurring species are distantly related) to phylogenetic clustering (i.e. co-occurring species are closely related) at larger spatial scales (Cavender-Bares *et al.* 2009; Kraft & Ackerly 2010). This happens, for example, when the competitive exclusion of closely related species, or the facilitation of distantly related ones, operates at smaller spatial scales than the filtering of closely related species by the environment. This scenario would increase PD values relative to the random assembly model at small scales, and decrease them at large scales, leading to a decrease of the slope of the observed PD–area curve compared with the null pattern. At spatial scales where dispersal limitation is a major driving force, evolutionary forces causing sister species to co-occur, such as *in situ* speciation, would result in a stronger signal of clustering compared with the null as spatial scale decreases. This situation would result in a steeper PD–area curve relative to the null.

Deviations from null phylogenetic beta-diversity patterns have been reported in the past, in particular for communities sampled along strong environmental gradients (Hardy & Senterre 2007; Bryant *et al.* 2008), or across sites separated by strong barriers to dispersal (e.g. mountain ranges, oceans or large geographic distances; Forest *et al.* 2007; Chave *et al.* 2007; Graham *et al.* 2009). We observed deviations from the random assembly hypothesis when comparing communities across Mediterranean-type ecosystems, reflecting the presence of distinct floras in regions that have been geographically separated over evolutionary time scales. The strength of the deviation corresponded to the degree of historical isolation and geological differences between regions. More generally, deviations from the random decay in phylogenetic similarity with geographic distance are likely to happen if geographic distance is associated with strong barriers to dispersal, or if species traits are evolutionarily conserved and geographic distance is strongly associated with environmental distance. In these cases, the spatial turnover of lineages will be faster than expected from species turnover alone, steepening the slope of the decay in phylogenetic similarity with geographic distance compared with the null.

In conclusion, we used information on the spatial distribution of species and a random sampling of phylogenies to develop the first sampling theory for spatial patterns of PD. This framework offers the promise of using, in future research, well-studied macro-evolutionary models of cladogenesis to understand how phylogenies map on ecological communities and the landscape. This may ultimately improve our ability to conserve biodiversity.

## SUPPORTING INFORMATION

**Appendix S1** Mediterranean flora data and phylogeny.

**Appendix S2** Random community assembly.

**Appendix S3** Species–PD relationship of the combined phylogeny and sensitivity analysis.

**Appendix S4** Statistical tests relevant to spatial phylogenetic diversity patterns and predictions.

**Appendix S5** Potential loss of PD with habitat loss in Mediterranean-type ecosystems.

**Appendix S6** A general relationship between the species–PD curve, the species–area curve and the PD–area curve.

**Appendix S7** The decay of phylogenetic similarity with geographic distance.

**Appendix S8** Specific phylogenetic resolutions.

As a service to our authors and readers, this journal provides supporting information supplied by the authors. Such materials are peer-reviewed and may be re-organized for online delivery, but are not copy edited or typeset. Technical support issues arising from supporting information (other than missing files) should be addressed to the authors.
