# Supplementary material for: Spatial patterns of phylogenetic diversity
Source: Ecol Lett. 2011 Feb;14(2):141–9. doi: 10.1111/j.1461-0248.2010.01563.x (PMC3047708; doi:10.1111/j.1461-0248.2010.01563.x)
Supplement: Supplementary file 1 [file ele0014-0141-SD1.pdf]

Spatial patterns of phylogenetic diversity

H. Morlon, D.W. Schwilk, J.A. Bryant, P.A. Marquet,
A.G. Rebelo,C. Tauss, B.J.M. Bohannan, J.L. Green

This document comprises the following items:

- **Appendix S1:** Mediterranean flora data and phylogeny
- **Appendix S2:** Random community assembly
- **Appendix S3:** Species-PD relationship of the combined phylogeny and sensitivity analysis
- **Appendix S4:** Statistical tests relevant to spatial phylogenetic diversity patterns and predictions
- **Appendix S5:** Potential loss of PD with habitat loss in Mediterranean-type ecosystems
- **Appendix S6:** A general relationship between the species-PD curve, the species-area curve, and the PD-area curve
- **Appendix S7:** The decay of phylogenetic similarity with geographic distance
- **Appendix S8:** Specific phylogenetic resolutions

Appendix S1: Mediterranean flora data and phylogeny

Data Presence/absence data for woody angiosperms in the mediterranean climate zone of Australia, California, Chile and South-Africa were recorded between April and December 2006 (Fig. S1). On each continent, thirty nested quadrats were sampled at the 2.5 x 2.5 m, 7.5 x 7.5 m and 20 x 20 m scales (120 quadrats total). Sampled quadrats were laid out along transects ranging between $(30°42'S, 115°31'E)$ and $(29°16'S, 115°06'E)$ in Australia, $(36°26'N, 118°44'W)$ and $(37°06'N, 119°25'W)$ in California, $(34°22'S, 71°18'W)$ and $(33°05'S, 71°09'W)$ in Chile, and $(33°55'S, 19°11'E)$ and $(32°27'S, 18°53'E)$ in South-Africa. Quadrats were separated by geographic distances ranging from 20 m (adjacent) to 170 km. Within each quadrat, presence/absence data were recorded at the 2.5 x 2.5 m, 7.5 x 7.5 m and 20 x 20 m scales (nested sampling). Data were recorded only at the 20 x 20 m scale in California. A Google Earth File comprising all our sampling sites is available in the online Supplementary Information.

All woody angiosperms were collected, with no size cut-off. Specimens were identified by expert botanists in each region. Sub-species were lumped, resulting in a total of 538 species encompassing 254 genera and 71 families. In Australia, species were identified with reference to specimens held by the WA Herbarium and Florabase (the online database of the Western Australia Herbarium, http://florabase.calm.wa.gov.au/). In California, we used the Jepson manual (Jepson, 1993). In Chile, we used the Flora Silvestre de Chile (Hoffman, 2005). In South-Africa, species were identified with reference to specimens held by the Compton Herbarium (http://posa.sanbi.org/searchspp.php); records were checked against the latest synonyms in the National Herbarium Pretoria Computerised Information System (PRECIS). Species from the Restionaceae and Bromeliaceae are not woody; nonetheless, several genera from these families include species which fill an ecological sub-shrub niche as persistent, shrubby perennials. Therefore, puya species (Bromeliaceae) were included in Chile. Due to the ambiguity in categorizing species from the Restionaceae, these species were collected by the South-African field

crew, but not by the Australian crew. Hence, the analyses in the paper include species from the Restionaceae in South-Africa, but not in Australia.

Phylogeny The phylogeny of the 538 species collected was constructed as specified in the main text. Thereafter, we term the phylogeny of all 538 species the "combined phylogeny", and we term the phylogenies of the species present in each dataset the "regional phylogenies". Phylogenetic data added to (or differing from) data given by the Phylomatic2 repository as of March 2010 are provided at the end of this document. A visual representation of the combined and regional phylogenies is shown in Fig. S2.

Appendix S2: Random community assembly

In Australia, Chile and South-Africa, we tested for potential deviations from the random assembly hypothesis in all 30 samples (90 samples total) at the 2.5 x 2.5 m, 7.5 x 7.5 m, and 20 x 20 m scales. In California, we only tested for deviations from the random assembly hypothesis at the scale where data was available (i.e. the 20 x 20 m scale). Following Webb *et al.* (2002), we ranked the PD observed in a sample containing S species within the PD of 1000 communities assembled by randomly sampling S species in each regional phylogeny. The significance of the deviation from the random assembly model was then obtained by dividing the rank of the observed PD by the number of observations (1001). A relative rank lower than 0.05 indicates that communities are significantly less phylogenetically diverse than expected by chance given their species richness (clustering). A relative rank greater than 0.95 indicates that communities are significantly more phylogenetically diverse than expected by chance given their species richness (overdispersion). With this level of significance, only few communities deviated significantly from the random assembly hypothesis (Fig. S3). There was a tendency for clustering in communities from the kwongan at the 7.5 m and 20 m scales, and a tendency for overdispersion in

Supplementary Figure 1: Overview of the location and spread of sampling sites. From left to right: mediterranean climate zone of Australia, California, Chile and South-Africa. Below: illustration of the nested sampling performed in each quadrat and each Mediterranean-type region except California.

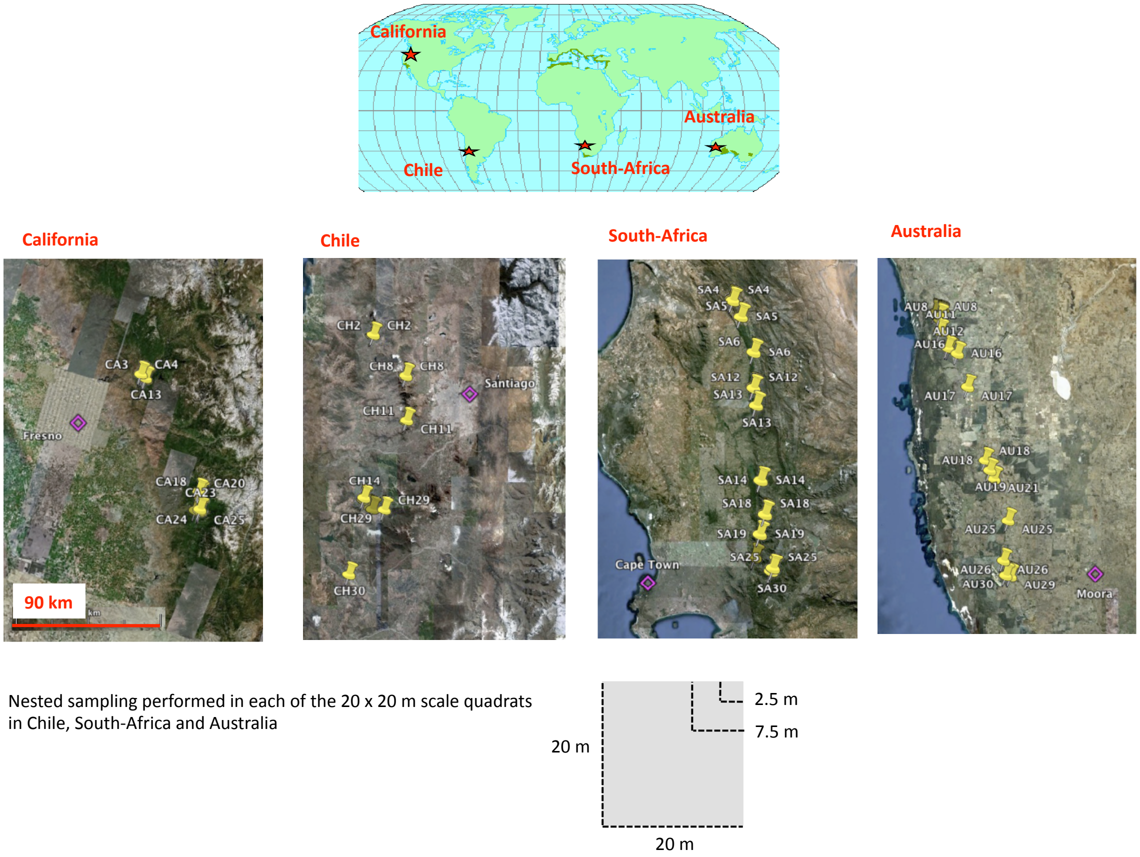

Supplementary Figure 2: Combined phylogeny (i.e phylogeny of all 538 species combined). In yellow: species collected in the kwongan (Australia). In red: species collected in the chaparral (California). In blue: species collected in the matorral (Chile). In green: species collected in the fynbos (South-Africa). Phylogeny plotted using iTOL (http://itol.embl.de/index.shtml).

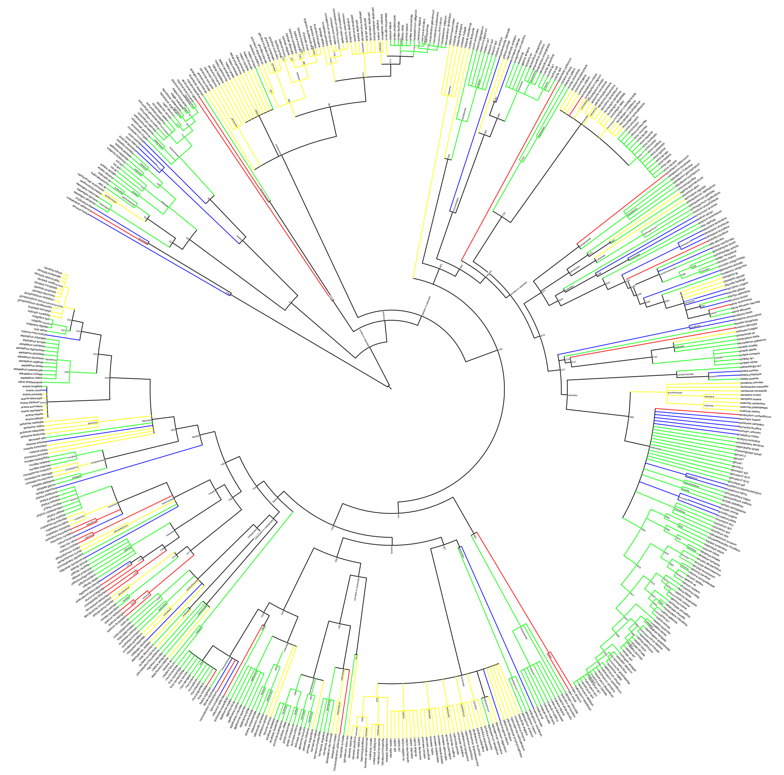

communities from the matorral and chaparral. These tendencies did not cause major deviations of the observed PD-area relationship from that expected under the random assembly hypothesis (Figure 3 from the main text, see also Appendix S4). Results obtained using other phylogenetic diversity metrics, namely the mean pairwise distance (MPD), which measures the mean phylogenetic distance among all pairs of species in the community and the mean nearest neighbor distance (MNND), which measures the mean phylogenetic distance to the nearest relative for all species in the community (Webb *et al.*, 2002), were qualitatively similar (results not shown).

Using a similar approach, we tested for potential deviations from the random assembly hypothesis across pairs of samples (within region), at the sample size used in the paper (i.e. the 20 x 20 m scale). We ranked the χ_{PD} observed between two samples, one containing S_1 species, the other containing S_2 species, and the two sharing $S_{1\cap2}$ species within the χ_{PD} of 1000 community pairs assembled by randomly sampling S_1 and S_2 species in each regional phylogeny while keeping $S_{1\cap2}$ constant. The significance of the deviation from the random assembly model was then obtained by dividing the rank of the observed χ_{PD} by the number of observations (1001). A relative rank lower than 0.05 indicates communities that are significantly less phylogenetically similar than expected by chance given the number of species present within each, and shared between, the two communities. A relative rank greater than 0.95 indicate communities that are significantly more phylogenetically similar than expected by chance given the number of species present within each, and shared between, the two communities. With this level of significance, only a few communities deviated significantly from the random assembly hypothesis, except in the fynbos, where there was a marked tendency for pairs of communities to be more similar than expected by chance (Fig. S4). This tendency caused the observed decay in phylogenetic similarity to lie above (i.e. have greater similarity values) the one expected under the random assembly hypothesis (Figure 4 from the main text, see also Appendix S4).

Our result that the PD supported by communities, and shared across communities, is most

Supplementary Figure 3: Random assembly within communities in the Mediterranean data. Histograms report the number of communities falling in a given rank class (relative rank as defined above). Communities falling on the left of the blue line are less phylogenetically diverse (i.e. have a smaller PD) than expected by chance given their species richness, and significantly so when they fall on the left of the first orange line. Communities falling on the right of the blue line are more phylogenetically diverse (i.e. have a higher PD) than expected by chance given their species richness, and significantly so when they fall on the right of the second orange line. From left to right: data collected at the 2.5 x 2.5 m, 7.5 x 7.5 m, and 20 x 20 m scales.

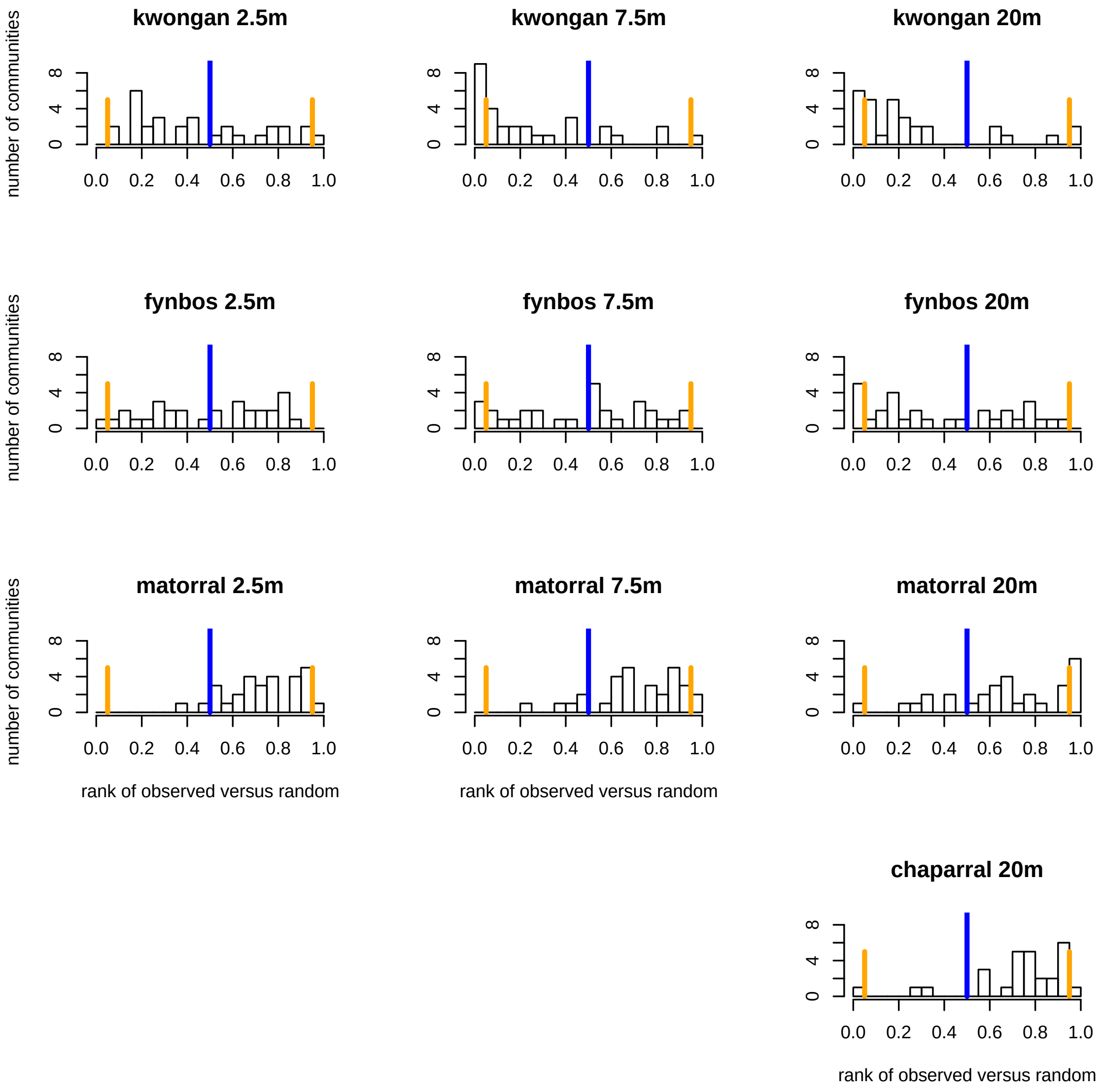

Supplementary Figure 4: Random assembly across communities in the Mediterranean data. Histograms report the number of community pairs falling in a given rank class (relative rank as defined above). Communities falling on the left of the blue line are less phylogenetically similar than expected by chance given their species richness and turnover, and significantly so when they fall on the left of the first orange line. Communities falling on the right of the blue line are more phylogenetically similar than expected by chance given their species richness and turnover, and significantly so when they fall on the right of the second orange line.

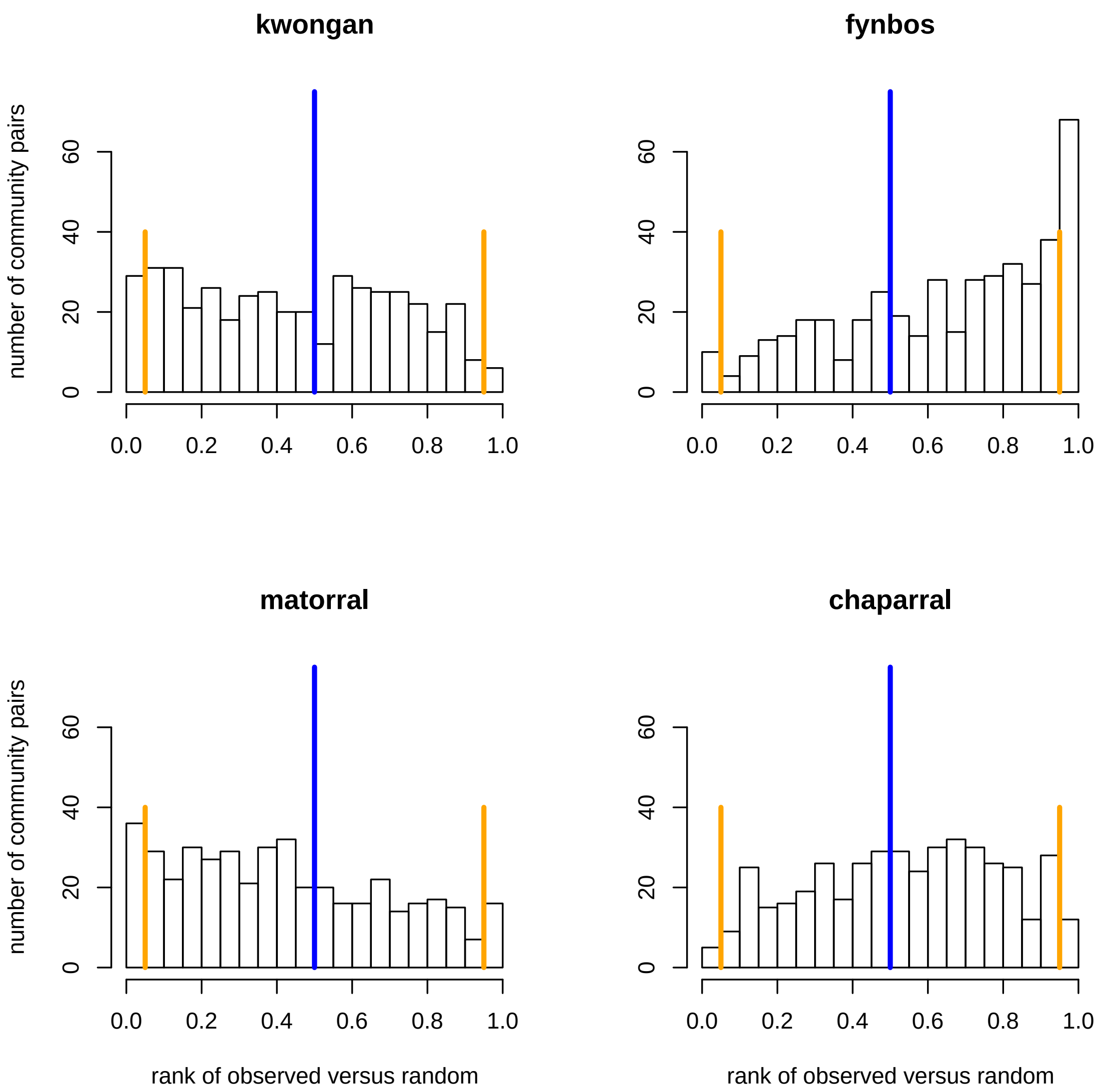

often not significantly different from expected under the random assembly hypothesis is conservative. Applying a Bonferroni correction in order to account for multiple testing (per continent, we performed 30 tests within communities, and 435 tests across communities tests) would reduce the number of communities or pairs of communities deviating significantly from this hypothesis.

Appendix S3: Species-PD relationship of the combined phylogeny and sensitivity analysis

Species-PD relationship of the combined phylogeny Fig. S5 illustrates the species-PD curve of the phylogeny of the 538 species combined, and how it compares to regional species-PD curves. The figure shows that the scale invariant species-PD curve holds on the combined phylogeny, with a z* exponent similar to exponents observed in regional phylogenies.

Sensitivity analysis To test the robustness of the species-PD curves and related exponents to uncertainty in the phylogeny, we separately explored the effect of polytomies and of the node age assignment algorithm. Code for these analyses is available at www.schwilk.org/research/data.html.

Polytomies An analysis of the effect of a lack of resolution on measurements of phylogenetic diversity, performed on simulated phylogenies, has shown that phylogenetic diversity is particularly sensitive to a lack of resolution basally (Swenson, 2009). Here, we were interested in the effect of missing resolution in our specific data, and on the specific patterns investigated in the paper (in particular the species-PD curve). To explore the effect of polytomies, we conducted the following procedure for each phylogeny (the combined phylogeny and the four regional phylogenies): 1) we created a set of 1000 alternative versions of the full unpruned phylogeny (i.e. angiosperm backbone tree + 538 species) and then ran the modified BLADJ algorithm on each of these to assign branch-lengths by dating undated nodes. For each of these

Supplementary Figure 5: Species-PD relationship of the combined phylogeny (in black) and comparison with the species-PD curve of each regional phylogeny. Data points (black circles) are the results of 100 simulated random samplings across the tips of the combined phylogeny. Lines are power-law fits across the data (data points corresponding to regional phylogenies not shown for clarity). The species-PD curve of the combined phylogeny is well approximated by a power-law curve with an exponent similar to those observed in regional phylogenies.

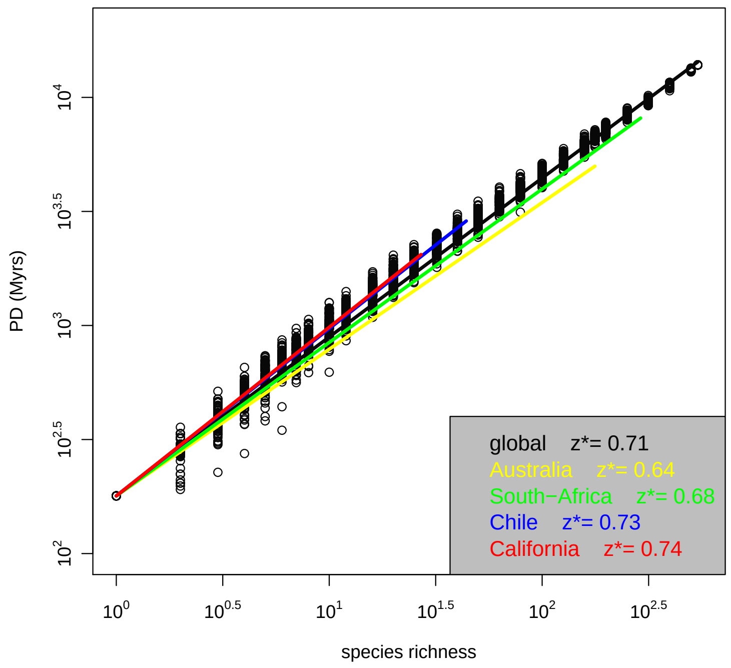

randomizations, we conducted a rarefaction analyses as described in the main text with 100 random draws for each species richness value.

The random resolution of polytomies followed by BLADJ branch-length assignment tended to curve the power-law species-PD curve downward towards the end of the sampling procedure (i.e. when almost all species were included), and consistently lowered z* values (Fig. S6). Randomly resolving polytomies pushed undated nodes towards the tips of the phylogenies. Deviations from the pattern observed without randomly resolving polytomies were the lowest in the Californian dataset where all nodes were resolved, and the largest in the Australian and South-African datasets where many polytomies remained. In the global phylogeny, the z* value was 0.71 without resolution, and the mean over 100 random resolutions was 0.65. In all phylogenies, the power-law approximation remained relevant after random resolution. In particular, the power-law always provided a better fit than the previously proposed logarithm (Nee & May, 1997) (black versus blue fit in Fig. S.6). Deviations from z* values obtained without random resolutions were always less than 0.1 unit. Hence, the presence of polytomies in the phylogenies does not compromise the main approach and conclusions of our study.

BLADJ branch-length assignment To test the sensitivity of the relationships to the BLADJ evenly-spaced node age method, we generalized the node dating algorithm to allow undated nodes to be assigned dates according to any normalized age distribution. We explored two variations.

In the first branch-length sensitivity analysis, instead of assigning node ages deterministically and evenly, node ages were assigned from a uniform random distribution with bounds set by fixed ages of ancestors and descendants. Using this method, we explored a set of 1000 phylogenies that varied in branch-length assignment for each topology. As expected, using a

Supplementary Figure 6: Robustness of the power-law shape of species-PD curves, and sensitivity of z* values, to random resolutions of polytomies followed by BLADJ branch-length assignment. For each dataset, we constructed 1000 randomly resolved phylogenies. On the left: data points (black circles) are the results of 100 simulated random samplings across the tips of one (randomly chosen) of the 1000 randomly resolved phylogenies. The black line is the power-law fit across the data points. The orange line is the power-law fit corresponding to the original unresolved phylogeny (data points not shown for clarity). This line is barely visible in the matorral and chaparral, because randomly resolving polytomies in the corresponding phylogenies had very little effect on the species-PD curve. Note that deviations from the orange line in the kwongan and fynbos do not reflect deviations from the power-law (deviations from the black line would), but rather deviations from the species-PD curve obtained without randomly resolving the polytomies. The blue line is the best-fit logarithm, shown for comparison with previous literature (Nee & May, 1997). The power-law (black line) provides a much better fit than the logarithm (blue line). On the right: Distribution of z* values for the 1000 randomly resolved phylogenies. The red line indicates the z* value corresponding to the original unresolved phylogenies. Randomly resolving polytomies pushed undated nodes towards the tips of the phylogenies, tended to curve the power-law species-PD curve downward towards the end of the sampling procedure, and consistently lowered z* values.

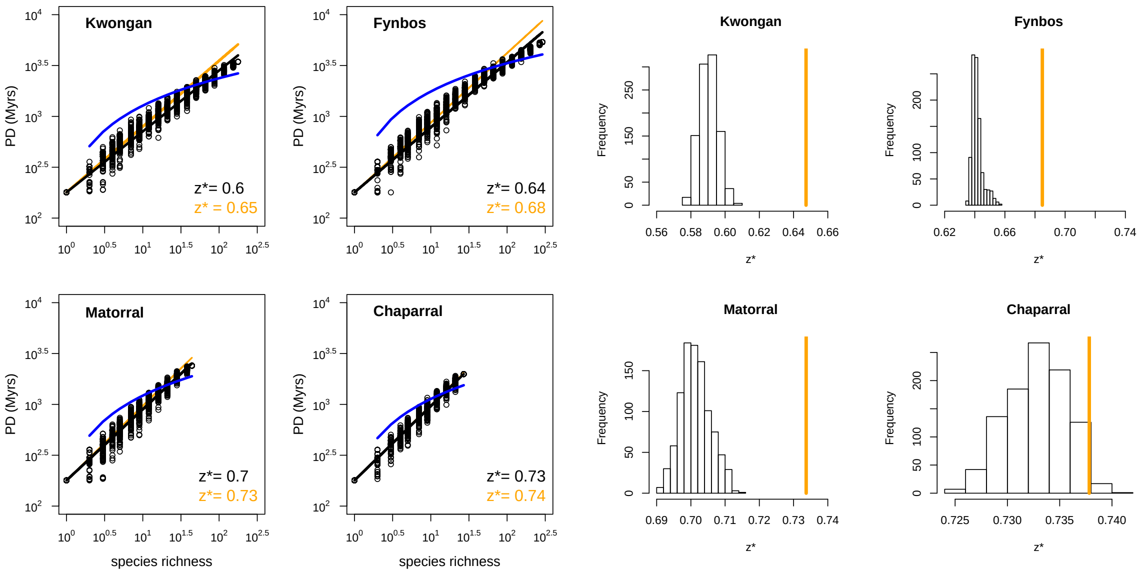

uniform distribution of ages instead of an evenly-spaced distribution did not change the shape of curve; it increased the variance in z* values, but did not drastically change mean values (Fig. S7).

Supplementary Figure 7: Robustness of the power-law shape of species-PD curves, and sensitivity of z* values, to branch-length assignment using a random uniform distribution of node ages instead of evenly spacing nodes. For each dataset, we constructed 1000 phylogenies with undated nodes assigned ages from a uniform distribution. On the left: data points (black circles) are the results of 100 simulated random samplings across the tips of one (randomly chosen) of the 1000 random phylogenies. The black line is the power-law fit across the data points. The orange line, which represent the the power-law fit corresponding to the original phylogeny, can barely been seen due to the robustness of the species-PD curve to the method of branch-length assignment. The blue line is the best-fit logarithm, shown for comparison with previous literature(Nee & May, 1997). On the right: Distribution of z* values for the 1000 random node age phylogenies. The red line indicates the z* value corresponding to the original phylogenies. Uniformly distributing nodes does not significantly change the shape of the species-PD curve, and does not greatly influence mean z* values. Rather, this method increases the variance in z* values.

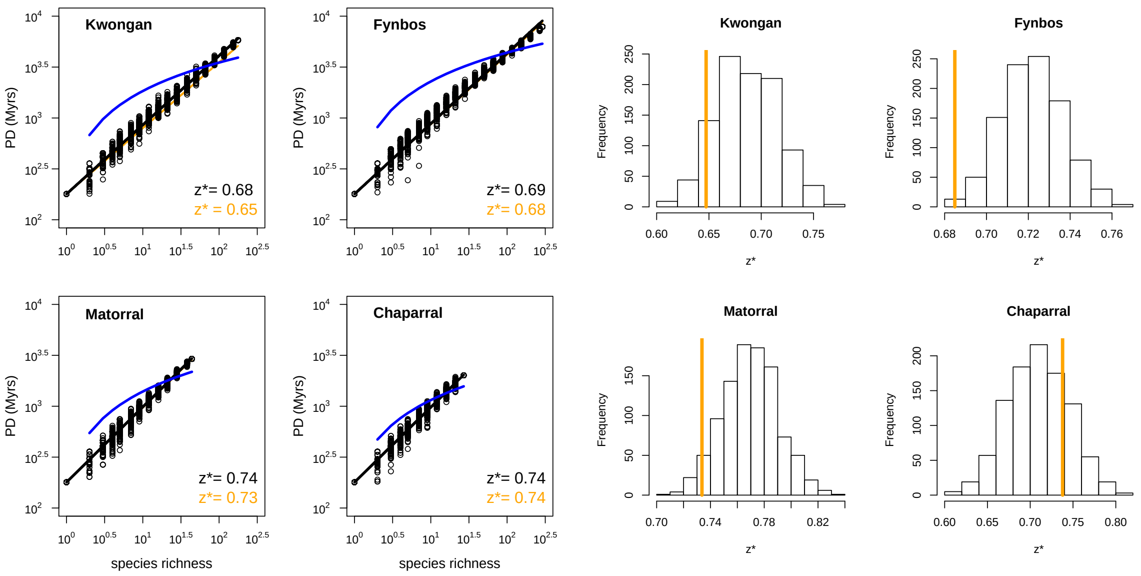

In the second branch-length sensitivity analysis, we explored node age distributions skewed towards either the root or the tips. For this analysis, node ages were drawn from a truncated

exponential distribution (again with bounds set by fixed ancestor and descendant ages). We defined a parameter, α, to control the amount of skewness. The truncated exponential parameter, λ, was then calculated from α: $\lambda = \frac{1}{\alpha}(A_a - A_d)$, where A_a and A_d are the ages of the fixed ancestor and descendant. Lower absolute values of alpha result in greater skew. We defined the algorithm such that λ can be relative to the ancestor age (positive alpha values, skewed toward root, resulting in longer branches at the tips) or the descendant age (negative alpha values, skewed toward tips, resulting in longer branches toward the root). We explored 1000 randomizations for each of six different truncated exponential distributions: three skewed towards the root with α values of 0.8, 0.6, and 0.2 and three skewed towards the tips with α values of -0.8, -0.6, and -0.2. We then ran the full rarefaction analysis on each of these phylogenies. The power-law shape of the curve was only affected for strong skews ($|\alpha|$=0.2) and more sensitive to a skew towards the tips than towards the root (Fig. S8). As expected, z* values decreased when nodes were distributed towards the tips, and decreased when nodes were distributed towards the root. Deviations from initial z* values were always less than 0.1 unit and did not compromise the main approach and conclusions of our study (Fig. S9).

Appendix S4: Statistical tests relevant to spatial phylogenetic diversity patterns and predictions

This section describes the statistical tests that we used to: 1) test the ability of the random assembly process to reproduce observed spatial phylogenetic diversity patterns (thereafter referred to as Test 1), and 2) test the accuracy of our spatial phylogenetic diversity theory predictions (Test 2). Test 1 is different from the tests performed in Appendix S2. In Appendix S2, we tested for potential deviations from the random assembly model at the level of individual communities (Fig. S3) or pairs of communities (Fig. S4). Here, we test for potential deviations from the random assembly model across communities, to assess if the observed spatial patterns deviate from

Supplementary Figure 8: Robustness of the power-law shape of species-PD curves to branch-length assignment using a distribution of node ages skewed towards the root or the tips instead of evenly spacing nodes. α values determine the direction and strength of the skew. Top row: distribution skewed toward the root (i.e. undated nodes are pushed towards the root). Bottom row: distribution skewed towards the tips (i.e. undated nodes are pushed towards the tips). From left to right: increasing skew. For each α value, we constructed 1000 phylogenies in which node ages were drawn from a truncated exponential distribution. The power-law shape is robust. Results - shown here for the combined phylogeny - were similar for regional phylogenies.

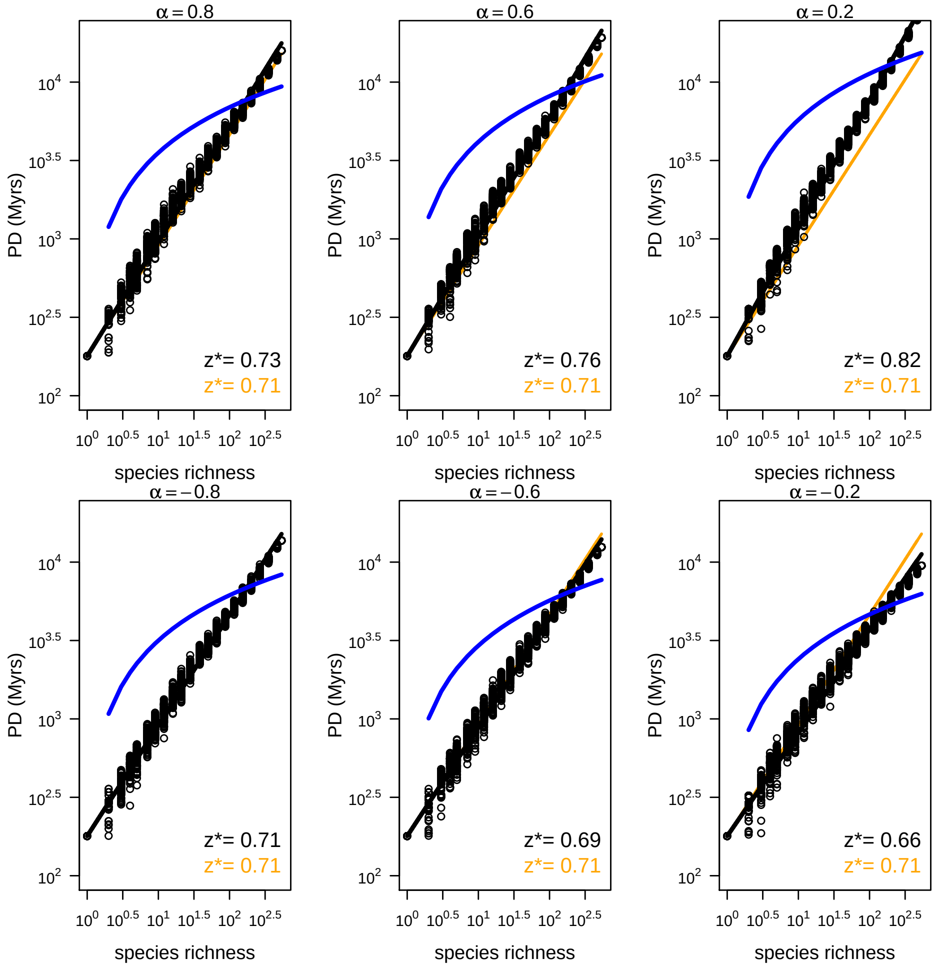

Supplementary Figure 9: Sensitivity of z* values to branch-length assignment using a distribution of node ages skewed towards the root or the tips instead of evenly spacing nodes. α values determine the direction and strength of the skew. Top row: distribution skewed toward the root (i.e. undated nodes are pushed towards the root). Bottom row: distribution skewed towards the tips (i.e. undated nodes are pushed towards the tips). From left to right: increasing skew. For each α value, we constructed 1000 phylogenies in which node ages were drawn from a truncated exponential distribution. As expected, phylogenies with longer terminal branch-lengths (top) have higher z* values, and phylogenies with shorter terminal branch-lengths (bottom) have higher z* values. Results - shown here for the combined phylogeny - were similar for regional phylogenies.

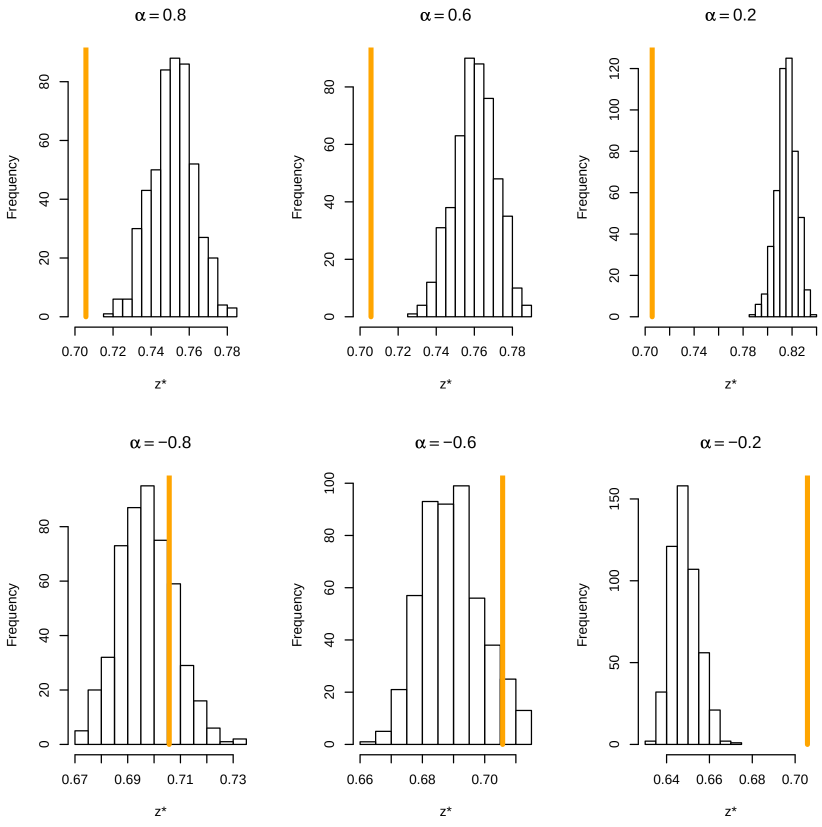

those expected under random community assembly. To perform Test 1, we compared observed curves to the 95% confidence envelopes of the curves obtained by simulations of the random assembly process (see below). To perform Test 2, we compared the predicted curves to these same confidence envelopes.

Statistical tests related to PD-area curves

To construct the 95% confidence envelope of the PD-area curve in a given Mediterranean region, we performed the three following steps: 1) we ran 1000 simulations of the random assembly process (keeping species richness constant) across all 30 samples at all scales, 2) we constructed the PD-area curve corresponding to each simulation by averaging, at each spatial scale, PD values across the 30 samples, and 3) we excluded, at each spatial scale, the 5% most extreme values.

To test the ability of the random assembly process to reproduce the observed PD-area curve (Test 1), we compared this observed curve (obtained by averaging across the 30 samples at each scale) to the 95% confidence envelope of the PD-area curve. The observed PD-area curve (orange line in Fig. S10) fell within the 95% confidence envelope of the curve obtained under random assembly (black lines, Fig. S10). In agreement with the results found in Appendix S2 (Fig. S3), the observed PD tended to be lower than expected under random assembly in the kwongan (specially at the largest spatial scales) and fynbos (at all spatial scaes), and higher than expected under random assembly in the chaparral. None of these tendencies were significant.

To test the accuracy of the PD-area predictions (Test 2), we compared the predicted curves (Equations 2 and 4 from the main text) to the 95% confidence envelope. The predictions from Equation 2 do not make the assumption that the species-area curve is power-law, whereas the predictions from Equation 4 do. The predictions from Equation 4 (blue dashed lines, Fig. S 10) were in good agreement with the predictions from Equation 2 (blue circles, Fig. S10),

demonstrating the validity of using the power-law to describe the species-area curve in our data. The predicted curves (blue circles and blue dashed lines) fell within the 95% confidence envelope obtained under random assembly (black lines, Fig. S10) in the kwongan and chaparral. In the fynbos, the predicted curves were close to the lower bound of the 95% confidence interval. This deviation was due in part to the fact that PD values were slightly lower than expected by chance, and it was also due to deviations from the power-law assumption for the species-PD curve in the fynbos. Regardless of these deviations, the predicted curves yielded a reasonable quantitative description of the data.

Statistical tests related to the decay in phylogenetic similarity with geographic distance

To construct the 95% confidence envelope of the phylogenetic distance-decay curve, we performed the three following steps: 1) we ran 1000 simulations of the random assembly process (keeping species richness and turnover constant) across all 435 sample pairs at the 20 x 20 m scale 2) we constructed the phylogenetic distance-decay curve corresponding to each simulation by pulling data points falling in 0.2 distance bins (on a log scale) 3) we excluded, in each bin, the 5% most extreme values.

To test the ability of the random assembly process to reproduce the observed phylogenetic distance-decay curve (Test 1), we compared this observed curve (obtained by pulling data points falling in 0.2 distance bins, orange line in Fig. S11) to the 95% confidence envelope of the curve obtained under random assembly (black lines in Fig. S11). This comparison shows that the random assembly process tends to overestimate similarity values in the kwongan, and underestimate them in the fynbos, although this discrepancy is only significant at the smallest spatial separation in the kwongan. The fact that observed similarity values tend to be higher in the fynbos than expected under random assembly is in agreement with the results found in Appendix S2 (Fig. S4).

Supplementary Figure 10: Test of theory predictions for the PD-area relationship. The observed relationship (orange line) is in good agreement with relationships obtained under random assembly (95% confidence interval represented by black lines). The predictions from Equation 2 (blue circles) fail in the fynbos, mainly due to deviations from the species-PD curve power-law assumption. The predictions from Equation 4 (blue dashed lines) fail in the fynbos due to the previous failure of Equation 2. PD-area curves were obtained by averaging the data over the 30 samples at each spatial scale.

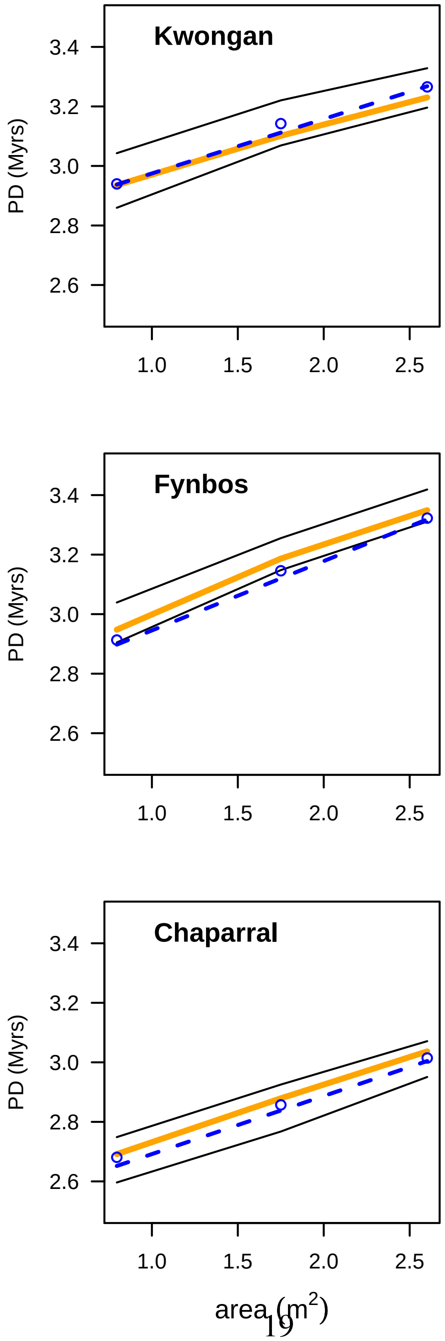

Deviations between the observed and predicted (Test 2, Equation 6 from the main text) phylogenetic distance-decay curves occurred in the kwongan and fynbos (Figure 4 from the main document). These deviations from the observed phylogenetic distance-decay curves have three potential origins: 1) deviations linked to the random assembly hypothesis 2) deviations linked to the power-law approximation for species-PD curves (resulting in Equation 5 from the main text) 3) deviations linked to fitting a logarithmic curve to the species distance-decay relationship (resulting in Equation 6 from the main text). The first source of error has been discussed in the previous paragraph, and explains part of the deviations in the fynbos. To evaluate the second source of error, we compared predictions given by Equation 5 (blue circles in Fig. S11) to the 95% confidence envelope of the curve obtained under random assembly (black lines). This comparison reveals that a good part of the deviations observed in the fynbos is linked to the power-law assumption for the species-PD curve, which results in a consistent underestimation of phylogenetic similarity values. To evaluate the third source of error, we compared predictions given by Equation 6 (blue dashed lines; this equation assumes a logarithmic fit to the species distance-decay relationship) to predictions given by Equation 5 (blue circles; this equation does not make any assumption on the shape of the species distance-decay relationship). This comparison reveals that a good part of the deviations observed in the kwongan is linked to the logarithmic assumption for the species distance-decay relationship. Regardless of deviations from the theory predictions, these predictions yielded a reasonable quantitative description of the data.

Appendix S5: Potential loss of PD with habitat loss in Mediterranean-type ecosystems

We investigated the potential loss of PD within each Mediterranean-type hotspot. This loss represents the loss of PD in the region under study: PD may be preserved elsewhere on Earth

Supplementary Figure 11: Test of theory predictions for the decay in phylogenetic similarity with geographic distance. The observed relationship (orange line) is in general in good agreement with relationships obtained under random assembly (95% confidence interval represented by black lines), except in the fynbos were communities tend to be more phylogenetically similar than expected under random assembly. The predictions from Equation 5 (blue circles) fail in the fynbos, due to deviations from the species-PD curve power-law assumption. The predictions from Equation 6 (blue dashed lines) fail in the fynbos due to the previous failure of Equation 5, and in the kwongan due to deviations from the logarithmic curve for the species distance-decay relationship. Distance-decay curves were obtained by pulling data points in 0.2 distance bins (on a log scale).

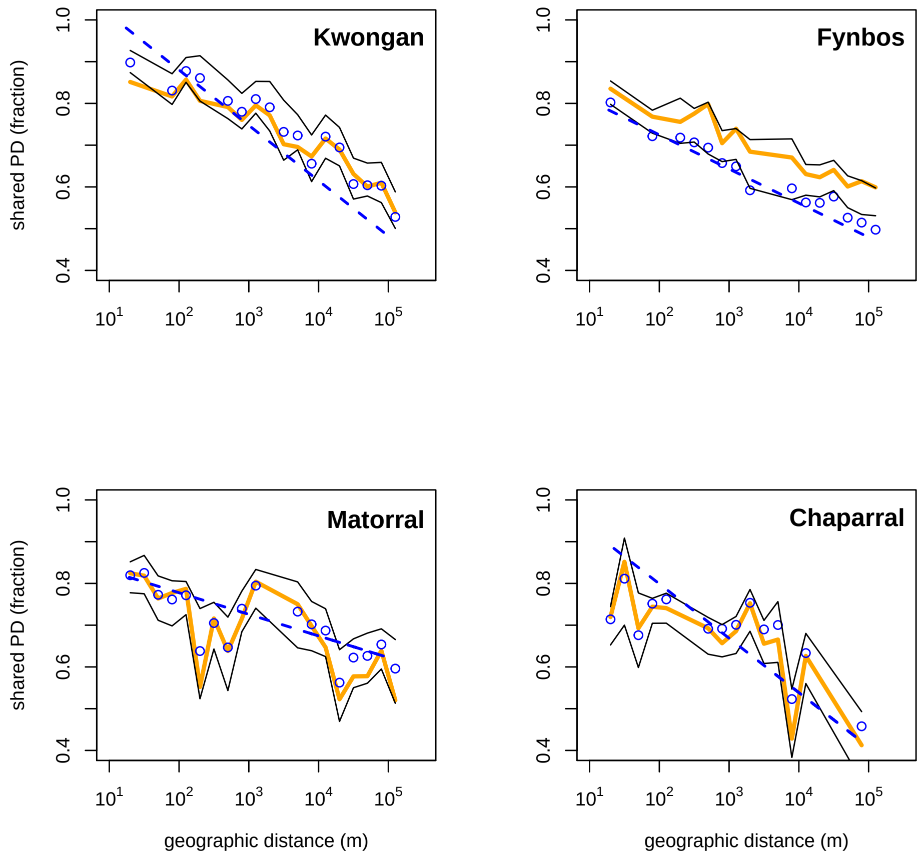

due to the presence of closely related species outside of the region considered. However, it is important to preserve PD at all spatial scales (see main text), and thus to investigate the potential regional loss of PD irrespective of what is preserved outside of the region.

By analogy with the classical species-area relationship (how species richness increases with area, Rosenzweig (1995)) which is commonly used to estimate the number of species threatened by habitat loss (Pimm *et al.*, 1995), we used the PD-area relationship to estimate the amount of PD threatened by habitat loss. Using the PD-area relationship to estimate PD loss presents several serious drawbacks in line with the drawbacks associated with using the species-area relationship to estimate species loss (Seabloom *et al.*, 2002). When full censuses, knowledge on species habitat, and information on habitat loss are available, more elaborate methods than species-area based methods exist for estimating diversity loss (Seabloom *et al.*, 2002; Faith, 2008). Area selection algorithms may also be used to select a set of protected sites maximizing the amount of PD preserved (Rodrigues & Gaston, 2002; Faith, 2006; Forest *et al.*, 2007). However, species-area based methods are useful when data on the systems to protect are incomplete, which explains that species-area curves are still used to derive estimates of species loss (e.g. Hubbell *et al.* (2008)).

In addition to limitations associated with using the species-area relationships, our estimates of PD-area slopes relied on small samples (i.e. the extrapolation is large), and they were based on data collected in one flora only, not the full hotspot. With these limitations in mind, we estimated how much floristic PD is potentially threatened in each Mediterranean-type hotspot as a result of habitat that has already been lost (i.e. due to extinctions that have already occurred or to an extinction debt), and how much is protected in current conservation areas. These calculations require an estimation of: 1) the scaling of PD with area, 2) the fraction of habitat lost relative to the total area of the hotspot, and 3) the fraction of habitat protected relative to the total area of the hotspot. The total area of the hotspot is defined as the sum of the biogeographic

region encompassing the current flora and the area that has been converted to human use.

To estimate the scaling between PD and area, we used the empirical data (Figure 3 in the main text). The slope of the power-law relationship between PD and area estimated in each continent was: 0.16 in Australia, 0.20 in Chile, and 0.23 in South-Africa. Due to a lack of nested data in California, we assumed the canonical value $z = 0.25$ for the power-law exponent of the species-area curve (Rosenzweig, 1995), and the empirical value $z^* = 0.74$ for the scaling of phylogenetic diversity with species richness.

To estimate the fraction of habitat lost and the fraction of habitat protected, we used recent estimates for the total, current and protected (defined as IUCN category I-IV) areas in each hotspot. For each Mediterranean-type region, these estimates were as follows (in the order: total, current, protected): Australia (D. Shepherd, personal communication updated from Beeston *et al.* (2006)): 297 928 km^2, 120 258 km^2 (40% of original), 37 844 km^2 (13% of original); California Mittermeier *et al.* (2005): 293 804 km^2, 73 451 km^2 (25% of original), 30 002 km^2 (10.2% of original); Chile (Wilson *et al.*, 2007; Mittermeier *et al.*, 2005): 148 383 km^2, 10 000 km^2 (7% of original), 1 332 km^2 (0.9% of original); South-Africa (Rouget *et al.*, 2006): 83 946 km^2, 57 923 km^2 (69% of original), 8 395 km^2 (10% of original). In the four regions combined, the estimated original, current and protected habitats span 824 061 km^2, 261 632 km^2 and 77 573 km^2, respectively.

Based on the slopes of the PD-area relationship and the fraction of area lost and protected, we estimated from Equation 4 (in the main text) the percentage of PD lost as a result of habitat loss in each Mediterranean-type region, yielding 14% in Australia, 23% in California, 42% in Chile, and 8.2% in South-Africa. The percentage of PD protected in conservation areas in each Mediterranean-type region was also predicted from Equation 4, yielding: 72% in Australia, 65% in California, 39% in Chile and 59% in South-Africa. To obtain similar estimates in the four regions combined, we used the average slope of the species-area relationships across con-

tinents (0.28), and the z* value for the combined species-PD curve ($z^* = 0.71$), resulting in a slope for the PD-area relationship of 0.20. If current habitat loss leads to extinctions predicted by the species-area relationship, then 20% of PD has been lost (or will likely be lost) in the Mediterranean-type ecosystems (excluding the Mediterranean Bassin), and 62% is protected in current conservation areas.

Appendix S6: A general relationship between the species-PD curve, the species-area curve, and the PD-area curve

In the main text, we used the power-law relationship to provide a simple characterization of the species-PD and species-area curves. The approach used, however, may be generalized to any functional characterization of the curves. Suppose that the species-PD curve follows any functional form f:

$$PD(S) = f(S) \tag{1}$$

Suppose that the species-area relationship follows any functional form g:

$$S(A) = g(A) \tag{2}$$

Under the hypothesis that communities are randomly assembled at each spatial scale, the expected PD contained in an area A ($PD(A)$) is the expected PD of the expected number of species contained in an area A. In other words:

$$PD(A) = PD(S(A)) = f(g(A)) = fog(A) \tag{3}$$

Hence, the functional form of the PD-area relationship is simply given by the composition of the species-PD curve with the species-area curve. Equation 3 may be used to predict, under the random assembly hypothesis, the shape of the PD-area relationship for any functional form of the species-PD and species-area relationships.

Appendix S7: The decay of phylogenetic similarity with geographic distance

The Sorensen index of similarity between two communities 1 and 2 is given by:

$$\chi = \frac{S_1 + S_2 - S_{1\cup 2}}{\frac{1}{2}(S_1 + S_2)} \tag{4}$$

where S_1 and S_2 represent species richness in community 1 and 2, respectively, and $S_{1\cup 2}$ represents species richness in community 1 and 2 combined. The expected similarity between two sampled communities spanning area A and separated by distance d is given by:

$$\chi(A, d) \sim \frac{2S(A) - S_{1\cup 2}(A, d)}{S(A)} = 2 - \frac{S_{1\cup 2}(A, d)}{S(A)} \tag{5}$$

A phylogenetic equivalent of the Sorensen index, measuring the fraction of phylogenetic branch-length shared between two communities 1 and 2, is given by (see Material and Methods in the main text):

$$\chi_{PD} = \frac{PD_1 + PD_2 - PD_{1\cup 2}}{\frac{1}{2}(PD_1 + PD_2)} \tag{6}$$

where PD_1 and PD_2 represent evolutionary history in community 1 and 2, respectively, and $PD_{1\cup 2}$ represents evolutionary history in community 1 and 2 combined. The expected phylogenetic similarity between two sampled communities spanning area A and separated by distance d is approximated by:

$$\chi_{PD}(A, d) \sim \frac{2PD(A) - PD_{1\cup 2}(A, d)}{PD(A)} = 2 - \frac{PD_{1\cup 2}(A, d)}{PD(A)} \tag{7}$$

Assuming that species are randomly assembled with respect to phylogeny and using the power-law scaling given by Equation 3 (from the main document) yields:

$$\chi_{PD}(A, d) \sim 2 - \Big(\frac{S_{1\cup 2}(A, d)}{S(A)}\Big)^{z^*} \tag{8}$$

Finally, combining Equations 5 and 8 yields Equation 5 in the main document. Note that Equation 8, and thus also Equation 5 in the main document, only holds when the area sampled are of the same size.

Under the logarithmic model $\chi(A,d) = \alpha + \beta \log_{10}(d)$, Equation 6 (in the main document) yields:

$$\chi_{PD}(A,d) \sim 2 - \Big(2 - \alpha + \beta \log_{10}(d)\Big)^{z^*} \tag{9}$$

$$\chi_{PD}(A,d) \sim 2 - (2-\alpha)^{z^*} \Big(1 + \frac{\beta}{2-\alpha} \log_{10}(d)\Big)^{z^*} \tag{10}$$

For $\frac{\beta}{2-\alpha} \log_{10}(d) << 1$, we obtain:

$$\chi_{PD}(A,d) \sim 2 - (2-\alpha)^{z^*} + \beta \frac{z^*}{(2-\alpha)^{1-z^*}} \log_{10}(d) \tag{11}$$

Appendix S8: Specific phylogenetic resolutions

Phylogenetic data added to, or differing from data given by the Phylomatic2 repository as of March 2010.

Asparagales Resolution as in apweb 2005, not apweb 2009, with Asphodelaceae sister to Xanthorrhoeaceae.

(orchidaceae,boryaceae,(blandfordiaceae,(lanariaceae,(asteliaceae,hypoxidaceae))),((ixioliriaceae, tecophilaeaceae),(doryanthaceae,(iridaceae,(xeronemataceae,((hemerocallidaceae,(xanthorrhoeaceae, asphodelaceae)),((alliaceae,(amaryllidaceae,agapanthaceae)),((hesperocallidaceae,aphyllanthaceae, (hyacinthaceae,themidaceae),agavaceae),(laxmanniaceae,(asparagaceae,ruscaceae))))))))));

Malvales Resolution as in apweb 2005, not apweb 2009, since apweb 2005 includes within family resolution for malvaceae, thymelaeaceae and dipterocarpaceae whereas apweb 2009 does not.

(neuradaceae,((gonystylus,(aquilaria,(daphne,(phaleria,dirca))))thymelaeaceae,(sphaerosepalaceae, ((bixaceae,diegodendraceae),cochlospermaceae),(cistaceae,(sarcolaenaceae,((dipterocarpus, (dryobalanops,(hopea,shorea,parashorea))),(vatica,cotylelobium),(upuna,vateria),anisoptera)dipterocarpaceae)), muntingiaceae,((((grewia,luehea),apeiba),(kleinhovia,byttneria)),((neesia,durio),(pentace,(heretiera,sterculia, scaphium),(tilia,pterospermum),ceiba)))malvaceae)));

Asteraceae Resolution as specified in Forest *et al.* (2007).

(corymbium, (chrysanthemoides, ((oedera, (((anaxeton, syncarpha), helichrysum), ((elytropappus, stoebe), metalasia))), (euryops, (othonna, senecio))), ((berkheya, cullumia), heterolepis)));

Fabaceae Resolution supplemented by resolutions specified in Forest *et al.* (2007).

((bauhinia, cercis)cercideae, (((((berlinia, brachystegia, oddoniodendron), brownea, cynometra, amherstia), ((hymenaea, guibourtia, peltogyne), tessmannia)), (barnebydendron, goniorrhachis), schotia, (colophospermum, prioria))detarieae, (((((dialium, martiodendron), petalostylis), apuleia), poeppigia)dialiinae, (((arcoa, ceratonia, gymnocladus, gleditsia)umtiza_clade, diptychandra, (((chamaecrista, cassia, senna)cassiinae, (((hoffmannseggia, zuccagnia), (caesalpinia, cenostigma, pomaria, poincianella, guilandia, stuhlmannia, haematoxylum, erythrostemon))caesalpinia_group, pterogyne)pterogyne_group), tachigali, ((conzattia, parkinsonia, peltophorum)core_peltophorum_group, ((mora, dimorphandra, erythrophleum)dimorphandra_group, (dinizia, pentaclethra, mimozyganthus, ((amblygonocarpus, adenanthera, tetrapleura, xylia, pseudoprosopis, calpocalyx)adenanthera_group,(piptadeniastrum, (entada, (plathymenia, ((neptunia, prosopis, prosopidastrum)prosopis_group, (desmanthus, leucaena)leucaenae_group, (dichrostachys, gagnebina)dichrostachys_group, (parkia, (microlobius, parapiptadenia, stryphnodendron, anadenanthera, pseudopiptadenia, adenopodia, piptadenia, mimosa)piptadenia_group, (acacia, ((faidherbia, zapoteca), lysiloma, enterolobium, albizia, ((chloroleucon, leucochloron,

blanchetiodendron)chloroleucon_alliance, (abarema, pararchidendron)abarema_alliance, (samanea, pseudosamanea)samanea_alliance, (havardia, ebenopsis, pithecellobium)pithecellobium_-alliance, (calliandra, cojoba, zygia, macrosamanea, cedrelinga, archidendron, inga)inga_alliance)) ingeae))))))))mimosoids)))), ((((bobgunnia, bocoa, candolleodendron, swartzia), ((ateleia, cyathostegia), trischidium))swartzieae, ((((myrocarpus, myroxylon, myrospermum), dussia), amburana), ((dipteryx, pterodon), taralea)), angylocalyx, (styphnolobium, pickeringia, cladrastis), (((uribea, calia), (holocalyx, lecointea, zollernia)), (luetzelburgia, sweetia), (((((((((arachis, stylosanthes), chapmannia), fiebrigiella), (brya, cranocarpus), platymiscium, grazielodendron, (cascaronia, geoffroea), (centrolobium, ramorinoa, inocarpus, tipuana, maraniona, pterocarpus, platypodium)), (discolobium, riedeliella)), (aeschynomene, machaerium, dalbergia, kotschya, (diphysa, (ormocarpopsis, ormocarpum), zygocarpum, pictetia), weberbauerella)),(adesmia, ((chaetocalyx, nissolia), (poiretia, (amicia, zornia)))))dalbergioids, (((marina, dalea), psorothamnus), (apoplanesia, ((amorpha, parryella), (eysenhardtia, errazurizia)))))amorpheae, (andira, hymenolobium), (vatairea, vataireopsis), ((((((((((genista, ulex), spartium), cytisus), (lupinus, anarthrophyllum)), dichilus), (crotalaria, lebeckia)), calpurnia), (piptanthus, (baptisia, thermopsis))),(((((templetonia, hovea), lamprolobium), (plagiocarpus, brongniartia)), harpalyce), cyclolobium, poecilanthe)), (bolusanthus, dicraeopetalum), (sophora, ammodendron, maackia), (diplotropis,(bowdichia, acosmium)), clathrotropis), ormosia)genistoids, (baphia, (((((aotus, gastrolobium), isotropis), gompholobium, daviesia, bossiaea)mirbelieae, hypocalyptus), (((((((((((macroptilium, mysanthus), (strophostyles, dolichopsis)), (oxyrhynchus, ramirezella), phaseolus, vigna, physostigma), (dipogon, lablab)), (spathionema, vatovaea), (((dolichos, nesphostylis), macrotyloma), sphenostylis), wajira), (cologania, (pseudovigna, neorautanenia), pueraria, amphicarpaea, (ophrestia, glycine), ((otholobium, psoralea), (rupertia, psoralidium, pediomelum)))), (erythrina, psophocarpus)), butea), ((campylotropis, desmodium), apios))phaseoloids, (abrus, (galactia, (philenoptera, (piscidia, ((lonchocarpus, dahlstedtia, deguelia, be-

haimia, bergeronia), (apurimacia, mundulea, tephrosia), (derris, paraderris), neodunnia, brachypterum, (millettia, pongamiopsis)))))), fordia, austrosteenisia, dalbergiella, xeroderris, platycyamus)millettioids, (phylloxylon, (indigofera, (cyamopsis, microcharis)))indigofereae), ((((anthyllis, hammatolobium, lotus, ornithopus), (coronilla, hippocrepis))loteae, (((((genistidium, peteria), coursetia), olneya, poissonia, sphinctospermum, robinia), (poitea, gliricidia)), (hebestigma, lennea))robinieae, (sesbania, (glottidium, daubentonia))sesbanieae), (wisteria, callerya, glycyrrhiza, ((((caragana, halimodendron), (alhagi, (hedysarum, onobrychis))), (oxytropis, astragalus, sphaerophysa, colutea, (lessertia, sutherlandia), (swainsona, (carmichaelia, clianthus)))), (parochetus, (galega, (cicer, ((((melilotus, trigonella), medicago), ononis),(trifolium, (vicia, (lens, (pisum, lathyrus))))))))))irlc))))))), (liparia, (rafnia, aspalathus)))))));

Iridaceae Resolution as specified in Forest *et al.* (2007).

((watsonia, bobartia), aristea, irid);

Lamiaceae Resolution as specified in Forest *et al.* (2007).

((stachys, teucrium), salvia);

Poaceae Elegia placed in the Restionaceae instead of the Poaceae, based on Forest *et al.* (2007).

(flagellaria, baloskion, (joinvillea, ((anomochloa, streptochaeta), (pharus, ((guaduella, puelia), ((((streptogyna, (ehrharta, (oryza, leersia))), ((pseudosasa, chusquea), (buergersiochloa, ((lithachne, olyra), (eremitis, pariana))))), (brachyelytrum, ((lygeum, nardus), ((melica, glyceria), (((diarrhena, (brachypodium, (avena, (bromus, triticum)))), ((phaenosperma, anisopogon), (ampelodesmos, (piptatherum, (stipa, nassella)))))))))bep, (micraira, (((chasmanthium, (thysanolaena, zeugites)), (gynerium, (danthoniopsis, ((miscanthus, zea), (panicum, pennisetum))))), (eriachne, (((aristida, stipagrostis), (merxmuelleraa, (danthonia, (karoochloa, austrodanthonia

)))), (((molinia, phragmites), (amphiopogon, arundo)), ((merxmuellerab, centropodia), ((pappophorum, (eragrostis, uniola)), (distichlis, (zoysia, (spartina, sporobolus))))))))))pacc))))));

Proteaceae Resolution as specified in Forest *et al.* (2007)

(bellendena, (placospermum, toronia), ((agastachys, symphionema), (eidothea, cenarrhenes, (stirlingia, (conospermum, synaphea)), franklandia, (aulax, petrophile), beauprea, (isopogon, (adenanthos, (leucadendron, protea)))), (carnarvonia, sphalmium, knightia, triunia, (neorites, orites), (helicia, hollandaea), lomatia, stenocarpus, (telopea, alloxylon, embothrium), (opisthiolepis, (buckinghamia, grevillea)), (banksia, (austromuellera, musgravea)), roupala, (lambertia, xylomelum), (macadamia, (brabejum, panopsis)), (cardwellia, (euplassa, gevuina)))));

Restionaceae Resolution as specified in Forest *et al.* (2007)

(willdenowia, (ischyrolepis, ((hypodiscus, elegia, thamnochortus), (staberoha, restio))));

Rutaceace Resolution as specified in Forest *et al.* (2007)

(((acronychia, flindersia), zanthoxlum), ((murraya, poncirus), ruta), agathosma, (adenandra, diosma));

Scrophulariaceae Resolution as specified in Forest *et al.* (2007)

(((pseudoselago, selago), microdon), oftia);

Thymelaeaceae Resolution as specified in Forest *et al.* (2007)

(gonystylus, (aquilaria, (daphne, (phaleria, dirca))), (gnidia, struthiola), (passerina, lachnaea));

Quercus Resolution as specified in Manos *et al.* (1999)

((quercus_kellogii, quercus_wislizeni), quercus_chrysolepis);

References

Beeston, G. R., Hopkins, A. J. M. & Shepherd, D. P. (2006). Land-use and vegetation in Western Australia. Tech. Rep. 249, Department of Agriculture, Western Australia.

Faith, D. P. (2006). *Actions for the 2010 biodiversity target in Europe - How does research contribute to halting biodiversity loss?* Oxford University Press, Oxford, pp. 70–71.

Faith, D. P. (2008). *Conservation biology: evolution in action*. pp. 99–115.

Forest, F., Grenyer, R., Rouget, M., Davies, T. J., Cowling, R. M., Faith, D. P., Balmford, A., Manning, J. C., Proches, S., van der Bank, M. *et al.* (2007). Preserving the evolutionary potential of floras in biodiversity hotspots. *Nature*, 445, 757–760.

Hoffman, A., J. (2005). *Flora Silvestre de Chile, Zona Central: Una guia para la identificacin de las especies vegetales ms frecuentes*. Cuarta Edicion, Princeton.

Hubbell, S. P., He, F., Condit, R., Borda-de Agua, L., Kellner, J. & ter Steege, H. (2008). How many tree species are there in the Amazon and how many of them will go extinct? *Proc. Natl. Acad. Sci.*, 105, 11498–11504.

Jepson (1993). *The Jepson manual: higher plants of California*. University of California Press, Berkeley.

Manos, P. S., Doyle, J. J. & Nixon, K. C. (1999). Phylogeny, biogeography, and processes of molecular differentiation in Quercus subgenus Quercus (Fagaceae). *Mol. Phyl. Evol.*, 12, 333–349.

Mittermeier, R. A., Gil, P. R., Hoffmann, M., Pilgrim, J., Brooks, T., Mittermeier, C. G., Lamoreux, J. & Da Fonseca, G. A. B. (2005). *Hotspots revisited: Earth's Biologically Richest and Most Endangered Terrestrial Ecoregions*. The University of Chicago Press, Chicago.

Nee, S. & May, R. M. (1997). Extinction and the loss of evolutionary history. *Science*, 278, 692–692.

Pimm, S. L., Russell, G. J., Gittleman, J. L. & Brooks, T. M. (1995). The future of biodiversity. *Science*, 269, 347–350.

Rodrigues, A. & Gaston, K. (2002). Maximising phylogenetic diversity in the selection of networks of conservation areas. *Biol. Conserv.*, 105, 103–111.

Rosenzweig, M. L. (1995). *Species diversity in space and time*. Cambridge University Press, Cambridge.

Rouget, M., Jonas, Z., Cowling, R. M., Desmet, P. G., Driver, A., Mohamed, B., Mucina, L., Rutherford, M. C. & Powrie, L. W. (2006). *The Vegetation of South Africa, Lesotho and Swaziland*. South African National Biodiversity Institute Pretoria, Strelitzia.

Seabloom, E. W., Dobson, A. P. & Stoms, D. M. (2002). Extinction rates under nonrandom patterns of habitat loss. *Proc. Natl. Acad. Sci.*, 99, 11229–11234.

Swenson, N. (2009). Phylogenetic resolution and quantifying the phylogenetic diversity and dispersion of communities. *PLoS ONE*, 4, e4390.

Webb, C. O., Ackerly, D. D., McPeek, M. A. & Donoghue, M. J. (2002). Phylogenies and Community Ecology. *Annu. Rev. Ecol. Syst.*, 475–505.

Wilson, K. A., Underwood, E. C., Morrison, S. A., Klausmeyer, K. R., Murdoch, W. W., Reyers, B., Wardell-Johnson, G., Marquet, P. A., Rundel, P. W., McBride, M. F. *et al.* (2007). Conserving biodiversity efficiently: what to do, where, and when. *PLoS. Biol.*, 5, 1850–1861.
